# Supplementary material for: A universal method for depositing patterned materials in situ
Source: Nat Commun. 2020 Oct 21;11:5334. doi: 10.1038/s41467-020-19210-0 (PMC7578796; doi:10.1038/s41467-020-19210-0)
Supplement: Supplementary file 1 — Supplementary information [file 41467_2020_19210_MOESM1_ESM.pdf]

Supplementary Information: A universal method for depositing patterned materials in-situ

Yifan Chen, Siu Fai Hung, Wing Ki Lo, Yang Chen, Yang Shen, Kim Kafenda, Jia Su, Kangwei Xia, Sen Yang

Contents

Supplementary Note 1: Material 3

Supplementary Note 2: Laser induced material deposition setup 3

Supplementary Note 3: Experimental Protocols 5

(1) Preparation of metallate and semiconductor nanoparticle reagents 5

(2) Laser induced material deposition 8

(3) Exposure parameters 8

(4) About the cleaning 9

Supplementary Note 4: Sample characterization 10

(1) Optical microscope imaging 10

(2) Electronic properties of metal and I-V curve measurement 10

(3) Effective resistivity estimation 13

(4) SEM and FIB measurements 15

(5) The energy-dispersive X-ray spectroscopy (EDX) 15

(6) Surface quality of the deposited Fe line 18

(7) Reduce the composition effect introduced by semiconductor nanoparticle 18

(8) Mechanical properties 23

(9) Spatial resolution 26

Supplementary Note 5: Study of the mechanism of the LIMD method 28

(1) Model of deposition 28

(2) Wavelength dependence 31

(3) Laser power and chemical concentration dependence 32

(4) Analysis of the fine structures inside the deposition 37

Supplementary Note 6: Diamond quantum sensing 38

(1) The LIMD and Diamond quantum sensing correlated setup 39

(2) Sensing the potential heating during the deposition process 40

(3) Characterization of the magnetic profile of deposited Ni micro-magnets 40

(4) Characterization of deposited Pt MW waveguides 43

Supplementary Note 7: Measurement on resistive flex sensor and touch sensor 45

(1) Resistive flex sensor 45

(2) Resistive touch sensor 46

Supplementary Note 8: Circuit board repairing 49

Supplementary Note 9: Reflow soldering 50

Supplementary References 51

Supplementary Note 1: Material

The chemicals used in the experiments are listed below:

Gold(III) chloride hydrochloride ($HAuCl_4$, 99.995%) Sigma-Aldrich

Zinc chloride ($ZnCl_2$, ≥98.0%) Tianjin Yongda Chemical Co.

Nickel chloride ($NiCl_2$, 98+%) Sigma-Aldrich

Chloroplatinic acid hydrate ($H_2PtCl_4 \cdot xH_2O$, ≥99.9%) Jiangsu Hanggui Catalyst Co.

Chloroplatinic acid hydrate ($H_2PtCl_6 \cdot xH_2O$, ≥99.9%) Jiangsu Hanggui Catalyst Co.

Silver nitrate ($AgNO_3$, 99.9999%) Sigma-Aldrich

Iron(III) chloride hexahydrate ($FeCl_3 \cdot 6H_2O$, ≥99%) Tianjin Guangfu Chemical Co.

Reduced graphene oxide powder (purity>99%) Hangzhou Hangdan optoelectronics technology Co.

Carbon inks:

Shanghai Hero Pen Company: No. 234 black ink

Sailor Pen: Nano Kiwa-guro Ink, ultra-black

Substrates:

Glass slides (25.0 × 75.0 mm 1.0-1.2 mm thickness from Lab'IN Co. & 22 × 22 mm 0.13 - 0.16 mm thickness from Paul Marienfield GmbH & Co. KG)

Quartz plate (20 × 20 × 1 mm) Lianyungang Weida quartz Co.

Sapphire plate (D8 × 1 mm) Shengyakang Optics co.

Indium tin oxide (ITO) plate (380 nm/1.1 mm thickness of ITO/glass) South China Science & Technology Company Limited

Kapton tape (50 μm thickness) Mileqi Adhesive Co.

Supplementary Note 2: Laser induced material deposition setup

In the experiment, a home-built direct laser writing system was used for the LIMD. The experimental setup is illustrated in Fig. S1. The laser in use is a continuous wave (CW) DPSS laser made by Laser Quantum, with a wavelength of 532 nm and a maximum power of 650 mW. An acousto-optic modulator (AOM) was used to switch the laser on and off with a power ratio beyond 60 dB.

The laser beam was reflected by a 532-nm notch filter. This allows most of the laser power to reach the sample while the transmission image can be captured by the CCD camera. A 4-f system was constructed with a two-axis Galvo scanner and two *f*=200 mm plano-convex lenses to form the scanning system. We used a Nikon 20x/0.75 air objective lens or a 100x1.3 N.A. oil objective lens on a piezo objective stage to focus the beam. Sample position was controlled manually by a 3-axis translation stage. A piezo objective stage, attached to the scanner, was used for fine adjustment of the focus.

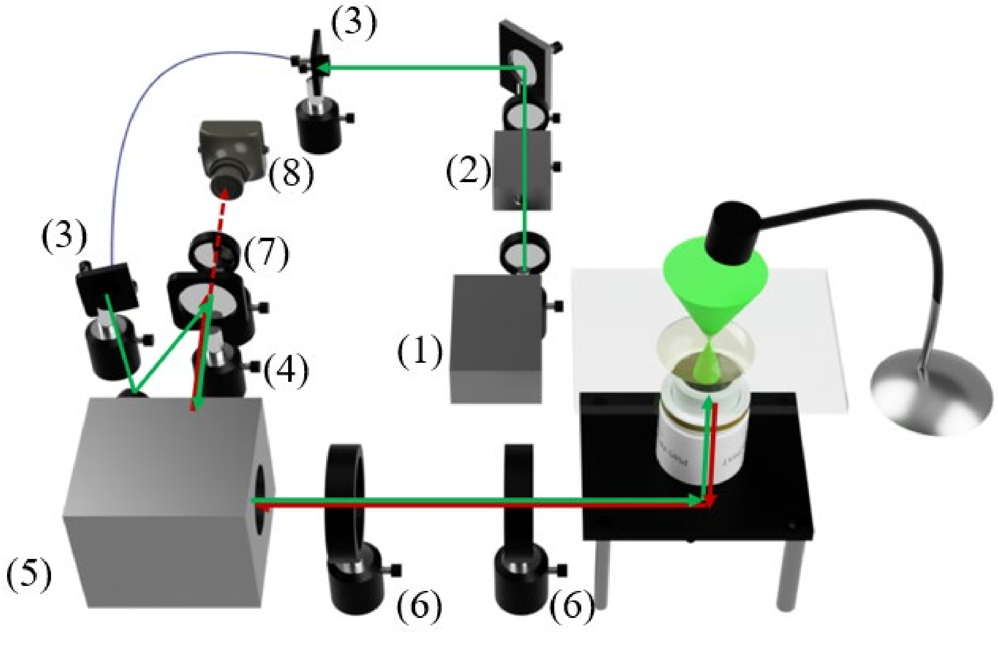

Figure S1. Overview of the home-built laser writing system. (1) A CW laser with 532 nm wavelength. (2) AOM. (3) Single mode fibre couplers. (4) Notch filter wavelength at 532 nm. (5) Two-axis Galvo scanner. (6) 200 mm focus length plano-convex lenses. (7) 100 mm focus length plano-convex lens. (8) CCD camera.

Above the sample, there was a white LED light for wide field imaging (see Fig. S2). The transmission image of the sample was imaged to a CMOS camera. The camera was used to check the focus and monitor the deposition process *in-situ*.

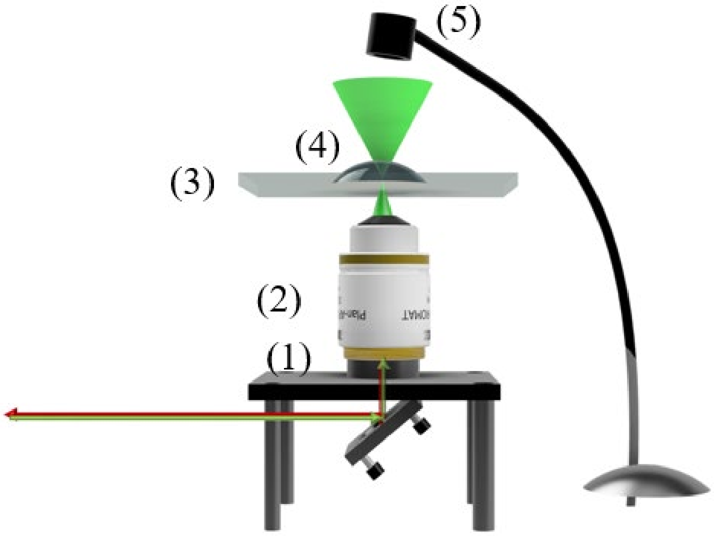

Figure S2. Sample and objective lens holders. (1) Piezo objective lens stage. (2) Air objective lens used in the experiments. (3) 3-axis translation stage. (4) Solution. (5) White light source.

Supplementary Note 3: Experimental Protocols

(1) Preparation of metallate and semiconductor nanoparticle reagents

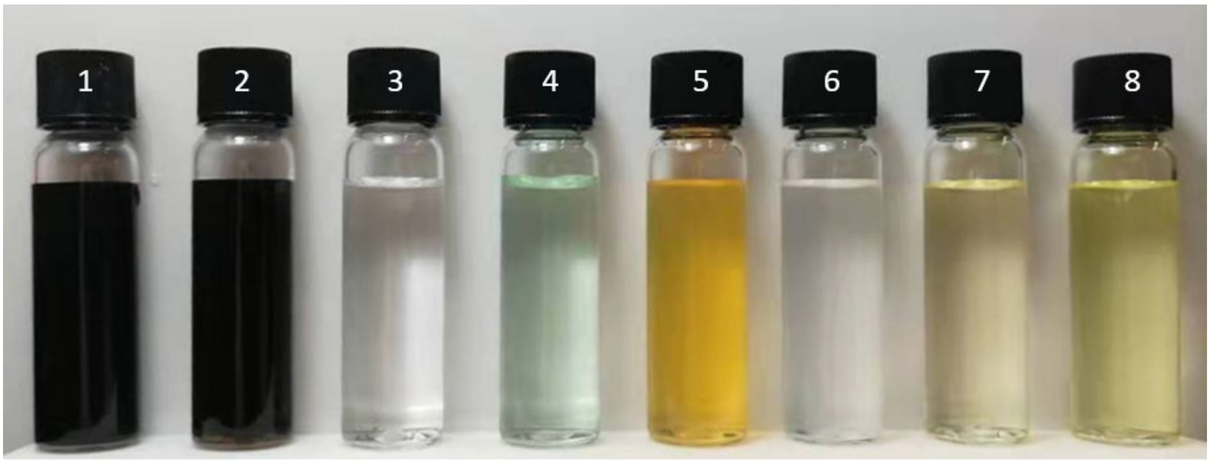

Figure S3 Prepared in-stock reagents. 1. Hero carbon ink. 2. Reduced graphene oxide solution. 3. Silver nitrate solution. 4. Nickel chloride solution. 5. Iron(III) chloride solution. 6. Zinc chloride solution. 7. Chloroplatinic acid solution. 8. Gold(III) chloride hydrochloride solution.

Part A: Gold(III) chloride hydrochloride ($HAuCl_4$), zinc chloride ($ZnCl_2$), nickel chloride ($NiCl_2$), chloroplatinic acid (H_2PtCl_6), silver nitrate ($AgNO_3$) and iron(III) chloride ($FeCl_3$) were dissolved in Milli-Q water separately, and the solutions were sonicated 15 mins to obtain different stock metal solutions. The optical images of the in-stock solutions are shown in Fig. S3. Their concentrations are the following: $AgNO_3$ 30 mmol/L, $NiCl_2$ 50 mmol/L, $FeCl_3$ 50 mmol/L, $ZnCl_2$ 200 mmol/L, H_2PtCl_6 30 mmol/L, $HAuCl_4$ 2.5mmol/L.

Part B: Reduced graphene oxide and carbon ink were dissolved in Milli-Q water separately, and the solutions were sonicated 20 mins to obtain different stock of reagents. The optical images of the in-stock solution are shown in Fig. S3. Their concentration is the following: 1. Carbon ink (from Hero Pen Company) 1:100. 2. Reduced graphene oxide 1mg/mL.

Dynamic light scattering (DLS) measurements of the reducing agents suggest that the average hydrodynamic diameters of graphene oxide and two types of carbon inks solution are 710 nm, 90 nm, and 160 nm respectively (see Fig. S4).

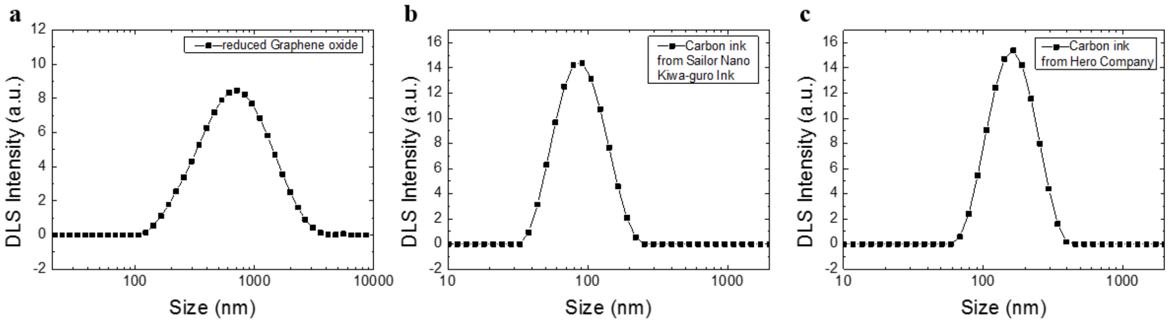

Figure S4. Dynamic light scattering (DLS) measurement of rGO and carbon ink solutions. Carbon ink solutions come from Sailor Pen: Nano Kiwa-guro Ink, ultra-black, and Shanghai Hero Pen Company: No. 234 black ink, respectively.

The final working concentration of 0.25-50 mmol/L was obtained after appropriate dilution with water. The mixture of different combination of metallate solutions and semiconductor nanoparticles were sonicated for 15 s before laser irradiation. The concentration of metallate solutions and semiconductor nanoparticles for material deposition on different substrates varies depending on the application. In Fig. S5 different metallate solutions mixed with carbon ink

are shown. The concentration of carbon ink in each solution is 1/6000. The concentrations of the metal ions are: $AgNO_3$ 7.5 mmol/L, $NiCl_2$ 12.5 mmol/L, $FeCl_3$ 12.5 mmol/L, $ZnCl_2$ 50 mmol/L, H_2PtCl_6 7.5 mmol/L, $HAuCl_4$ 0.625 mmol/L. In Fig. S6 different metallate solution mixed with reduced graphene oxide is shown. The concentration of reduced graphene oxide in each solution is 0.25 mg/mL. The metal ion concentrations are: $AgNO_3$ 7.5 mmol/L, $NiCl_2$ 12.5 mmol/L, $FeCl_3$ 12.5 mmol/L, $ZnCl_2$ 50 mmol/L, H_2PtCl_6 6.25 mmol/L, $HAuCl_4$ 0.625 mmol/L.

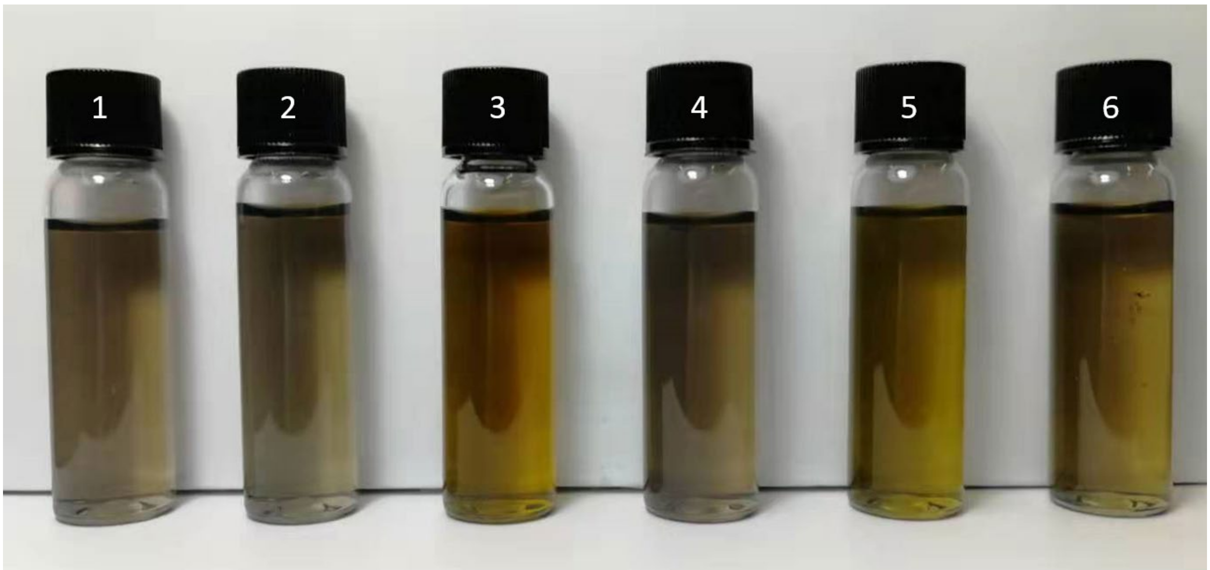

Figure S5 Metal solution mixed with carbon ink. 1. Silver nitrate solution. 2. Nickel chloride solution. 3. Iron(III) chloride solution. 4. Zinc chloride solution. 5. Chloroplatinic acid solution. 6.. Gold(III) chloride hydrochloride solution.

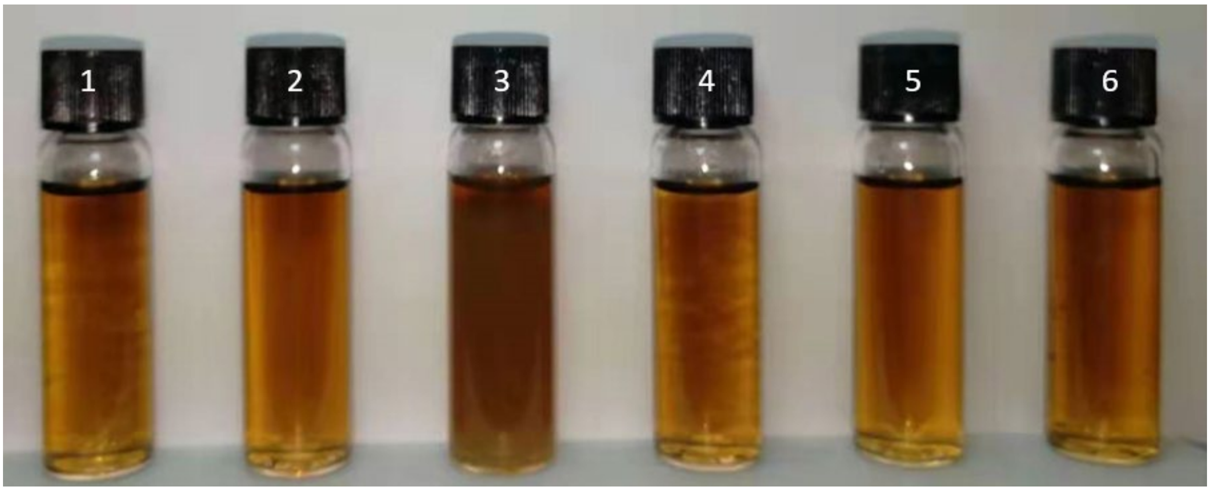

Figure S6 Metal solution mixed with reduced graphene oxide. 1. Silver nitrate solution. 2. Nickel chloride solution. 3. Iron(III) chloride solution. 4. Zinc chloride solution. 5. Chloroplatinic acid solution. 6. Gold(III) chloride hydrochloride solution.

(2) Laser induced material deposition

In the following, the deposition process of platinum on glass is described. A glass substrate covered with an aqueous solution of the mixture of H_2PtCl_6 and carbon ink was subjected to the laser writing system. A CW laser beam was focused at the substrate/liquid interface to introduce the reduction reaction between metallate and semiconductor nanoparticles as well as induce optical trapping. The laser on/off status was controlled by an AOM, while the transmission, and luminescence/scattering at the laser focus were imaged by the same microscope.

The LIMD parameters for large structures were the following: The exposure laser power was 60.4 mW measured in front of the objective lens. The exposure time per pixel was 30 ms, and the pixel size was 1.17 μm. For microstructure deposition (Fig. 3(a), (b)), the exposure parameter was 0.485mW power and 1s exposure time. After the exposure, the residue solution was taken out by a pipette. Further cleaning was performed by adding and taking out pure water solution 2~3 times. All the procedures had been done without taking out the sample from the LIMD system. When deposition of a second type of material is needed, the procedure, described above, can be repeated using a different solution.

(3) Exposure parameters

The exposure parameters in the experiments (The laser power refers to the power measured in front of the objective for metal deposition) are the following:

Parameters for thin Fe oxide line (Fig. 3(a) in the main text): Solution: 25 mmol/L $FeCl_3$ solution, and 200 times diluted hero ink were mixed by 1:1 volume ratio. The mixture was sonicated for 15s. Exposure parameters: 0.485mW laser power, 100 magnification NA=1.3 oil objective lens, exposure time 1s per pixel, step size 0.35, pixel size 446 nm.

Parameters for metal microstructure for conductivity and I-V curve measurement: The receipt for each metal was as following: 15 mmol/L H_2PtCl_6, 1.25 mmol/L $HAuCl_4$, 50 mmol/L $FeCl_3$, 100 mmol/L $ZnCl_2$ and 100 mmol/L $NiCl_2$. These metal solutions were then mixed with 3000 times diluted Sailor Nano Kiwa-guro ink by 1:1 volume ratio. All mixtures were sonicated for 15 s. Exposure parameter: 57.6mW laser power, 20x N.A.=0.75 objective lens, exposure time 30ms per pixel, step size 0.15, pixel size 1.17μm.

Parameters for element alphabet: The parameters were the same as those used for conductivity and I-V curve measurement. For the Ag alphabet, 7.5 mmol/L AgNO3 was mixed with 3000 times diluted Sailor Nano Kiwa-guro ink by 1:1 volume ratio and sonicated for 15s. Exposure parameter: 57.6mW laser power, 20x N.A.=0.75 objective lens, exposure time 30ms per pixel, step size 0.15, pixel size 1.17μm.

Parameters for Fig. 1 (c) in the main text: the parameters for metal deposition in the pictures were the same as those used for conductivity and I-V curve measurement correspondingly.

Parameters for Fig. 4 (i) in the main text: 25mmol/L $FeCl_3$ was mixed with 3000 times diluted Sailor Nano Kiwa-guro ink by 1:1 volume ratio and sonicated for 15s. The exposure parameters for the 3D topographic map were 20x N.A.=0.75 objective lens, exposure time 10 ms per pixel, step size 0.15, pixel size 1.17μm. And the laser power used from the lowest layer to the highest layer was 30mW, 35.4mW, 41.4mW and 47.4mW respectively.

(4) About the cleaning

Cleaning/changing solution without removing the sample is one of the uniqueness of the LIMD technique. Compared to the conventional photoresist-based lithograph technique, the LIMD doesn’t require a special treatment of the reagent, such as spin coating/soft baking/post baking. As shown in Fig. S7, after the material deposition, the residual reagent is simply taken out by the pipet. The sample is washed several times with DI water by the procedure of drop

cast and taken out with the pipet. The whole procedure has been done without taking out the sample from the laser writing system. When the deposition of a second material is required, we simply drop cast the new type of the reagent on the sample and continue the material deposition. It indicates that the pattern of different material doesn't require additional alignment.

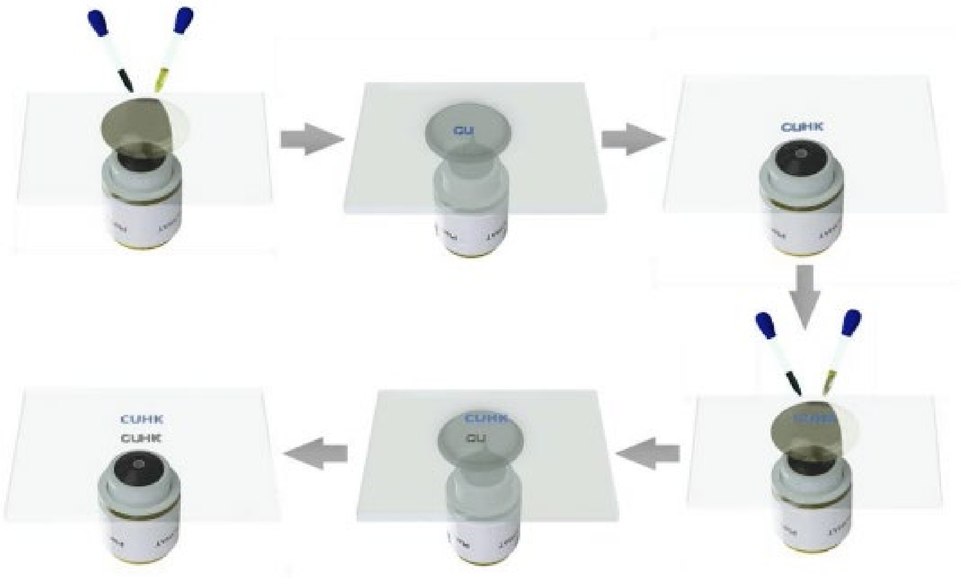

Figure S7. Procedure of multi-material deposition. After the first deposition, the solution is removed. The sample has been washed several times with DI water. After the cleaning process, the second solution is drop cast on the sample. The whole procedure has been done on the laser writing system without moving the sample.

Supplementary Note 4: Sample characterization

(1) Optical microscope imaging

Optical microscope images shown in Fig. 1(c) in the main text were acquired by a reflection type microscope. The microscope system consists of an Olympus BX60 Microscope with 5×, 10×, 20×, 50× and 100× UMPlanF objectives and a ZEISS AxioCam MRc5 microscope camera, which is controlled by AxioVision 4.0 software for image acquisition.

(2) Electronic properties of metal and I-V curve measurement

For electronic property study, different metals were deposited on a glass slide in the shape of 35 μm×350 μm lines. Silver glue (EPO-TEK H20E) was used to form contact electrodes. Two probing stages and a Keithley 2400 Source Measure Unit (SMU) multimeter were used for resistance and I-V curve measurements. The sample was monitored under the microscope when probes were moved in to form contacts onto the two electrodes. The I-V curves of the deposited Ni, Zn and gold microstructures are plotted in Fig.S8-10.

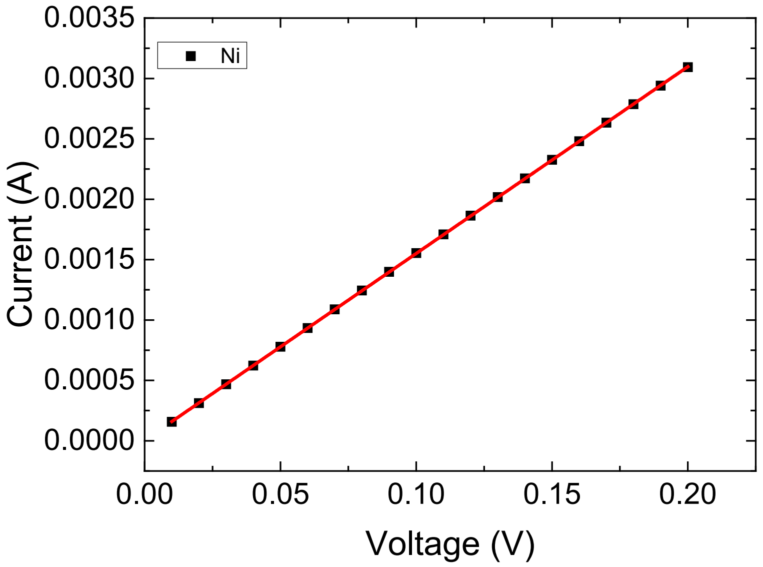

Figure S8. I-V curve of the deposited Ni microstructure.

Keithley 2400 was first used in Ohm measurement mode to check whether the sample is insulating or conductive. Then it was set to constant voltage output mode and connected to a computer via a GPIB to USB connector for data acquisition by instrument driver in LabView. For Pt and Ni sample (resistance lower than 100 Ohm), the voltage was scanned from 1 to 20 mV in 1 mV step to avoid strong heating of the sample. For Zn, Fe and Au samples (Zn resistance over 10 kOhm and Fe and Au above 200 MOhm measurement range), the voltage was scanned from 1 to 20 V to ensure, that the detected current is still well above the measurement limit of 10 pA[1].

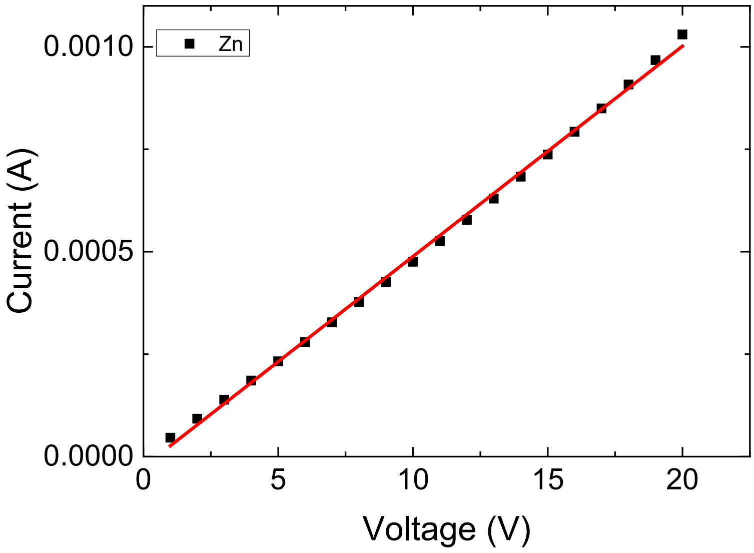

Figure S9. I-V curve of the deposited Zn microstructure.

For data shown in Fig. 3 (e), (f) in the main text: a set of Pt was deposited on glass slides as 23.4 μm×702 μm lines using 30ms exposure time and 57.6mW power. It has 6 lines. Then, half of them were sintered by the laser after the deposition and cleaning and the other half were left for comparison. The conductivity of all structures was measured. Then the shape was examined under a scanning electron microscope (SEM) and focused ion beam (FIB) machine. Under SEM, the length and width of each Pt line were measured individually. Next, the FIB was used to cut a cross section and the height was determined by measuring the height at different positions along the cross section and taking the average value. The conductivity of the Pt sample can be calculated from its resistance, length, width, and height. Besides the effect of sintering, the reproducibility of Pt performance could be learned from a comparison among samples of the same deposition parameters.

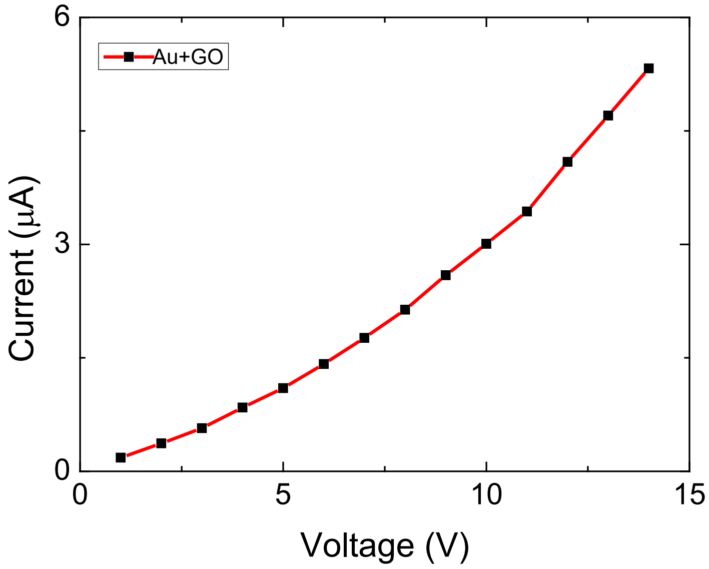

Figure S10. I-V curve of the deposited gold and rGO microstructure.

Table 1. Details of the deposited Pt microstructures.

Treatment	Sample	Resistance Ω	Length/μm	Width/μm	Height/μm	Conductivity/%
Not Sintered	1	15.77	582.8	23.65	1.707	9.70
	2	16.98	582.8	22.35	1.333	12.21
	3	13.19	632.2	23.65	2.109	10.18
Sintered	1	19.93	624.4	23.02	0.771	18.72
	2	16.33	616.7	23.38	1.169	14.65
	3	12.80	481.0	21.99	0.964	18.80

Table1 is a summary of the Pt conductivity measurement data.

(3) Effective resistivity estimation

The deposition is made of both carbon particles and Pt layers. To estimate the effective resistivity of the material we fabricated, here we use the following model. Assuming the material deposited consists of multiple square cylinder units, that are in close contact and each unit is made up of a carbon square cylinder core surrounded by platinum layer (Fig. S11(a)). From the EDX (Energy-dispersive X-ray spectroscopy) data of sintered Pt line for conductivity measurement, the mass percentage of the element carbon is 6.61%. The density of carbon is assumed to be 2g/cm^3 and Pt to be 21.45g/cm^3 and the side length ratio of the central carbon

core a/A can be deduced, where a and A are half the length of carbon core's sides and platinum layer's sides respectively (Fig. S11(b)).

If we inject the current from left to right along the x direction and assuming carbon and platinum are in parallel relation along y direction but in series relation with themselves along x direction, we can get an equation describing the effective resistivity as follows,

$$\int_0^A \frac{\rho\, dx}{A} = \int_a^A \frac{\rho_p\, dx}{A} + \int_0^a \frac{\frac{\rho_c\, dx}{a} \frac{\rho_p\, dx}{(A-a)}}{\frac{\rho_c\, dx}{a} + \frac{\rho_p\, dx}{(A-a)}} \quad (1)$$

where ρ is the effective resistivity of our sample, ρ_p and ρ_c are the resistivity of platinum and carbon respectively, a and A are the same as above, the x and y axis can be inferred from Fig. S11(b). The limits of integrals in Eq. (1) are reduced because of symmetry. The resistivity of carbon is chosen to be $35\mu\Omega \cdot m$ and Pt to be $0.106\ \mu\Omega \cdot m$. We calculated the resistivity ρ is $0.221\ \mu\Omega \cdot m$, which means its conductivity is 42.5% of bulk Pt. This is a theoretical upper limit for considering carbon as the only influencing factor for conductivity. Due to the imperfectness in both interfaces among pillars and on the upper surface of the structure, the real conductance is lower. Shown in Fig. 3 in the main text, the conductance is 18.7% of bulk Pt, i.e. 44.0% of the theoretical upper limit.

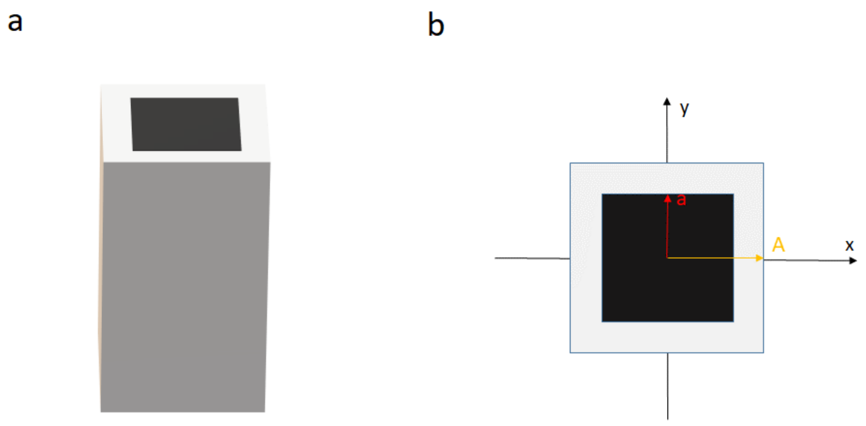

Figure S11: a, The square cylinder model. b, The cross section of the square cylinder.

(4) SEM and FIB measurements

Scanning electron microscope (SEM) is a powerful tool to characterize the performance of the deposited structures. Samples have first sputtered a thin layer of gold ~20 nm in the sputtering machine (POLARON SC502 Sputter Coater) to increase the conductivity for the electron beam. The FEI (ThermoFisher Scientific) Scios2 Dual Beam was used to perform SEM and FIB. For all the SEM image acquisition, the acceleration voltage was 5 kV.

For milling of I-V curve sample to measure its height and observe the quality of its cross-section, the acceleration voltage was 30 kV, an ion beam (Si) of large current 7 nA was first applied to expose the cross section and then an ion beam of lower current 0.3 nA cleaned the milling residue on the cross section. For milling of Fe thin line, the acceleration voltage was 30kV, an ion beam of low current 0.3 nA was applied and no cleaning was performed, because the sample size was submicron and an ion beam of low current applied to a small area would not leave much residue on the cross section. Fig. S12 shows an example of the cross section of the deposited Fe line.

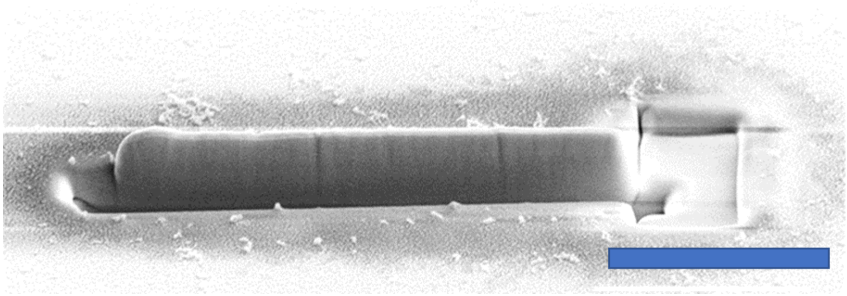

Figure S12: The cross section of the deposited iron line shown in Fig. 3(a) in the main text. The blue scale bar is 5 um.

(5) The energy-dispersive X-ray spectroscopy (EDX)

FEI SEM QF400 equipped with an energy-dispersive X-ray (EDAX. Inc.) spectroscopy was used to identify the elements in the deposition layer. The EDX mapping of deposited

materials is shown in Fig. S13-18. We can clearly see the peaks of the target deposition elements. In every EDX spectrum, there are also signals related to the presence of C, Si, and O elements, coming from carbon based nanoparticles and glass cover slides. It is worth noting that in Fig. S15 the EDX spectrum of the deposited iron structure shows a strong oxygen peak. It indicates that iron was oxide in the solution during the LIMD.

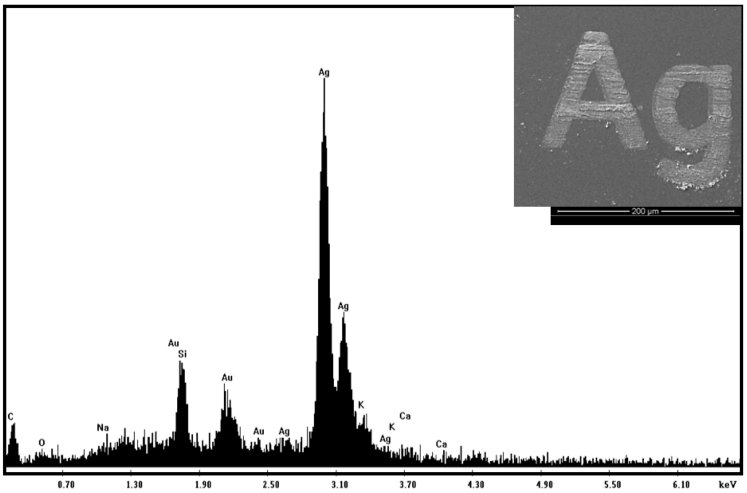

Figure S13. EDX of the deposited silver structure.

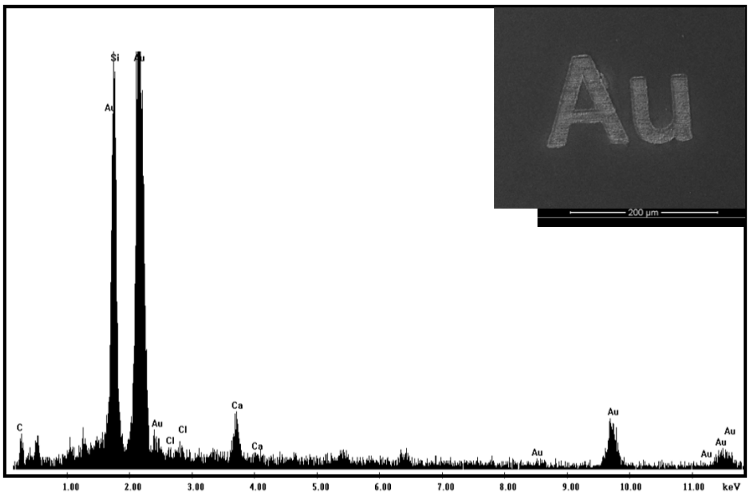

Figure S14. EDX of the deposited gold structure.

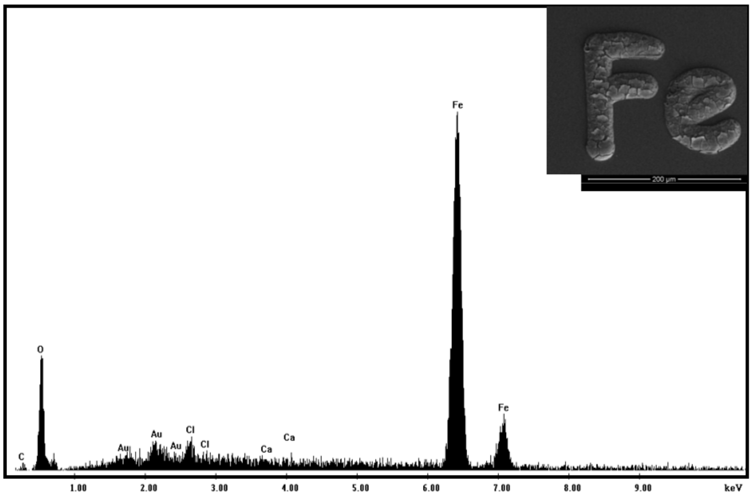

Figure S15. EDX of the deposited iron structure. The oxygen peak, showing up, indicates, that iron oxide has been present in the solution during the deposition.

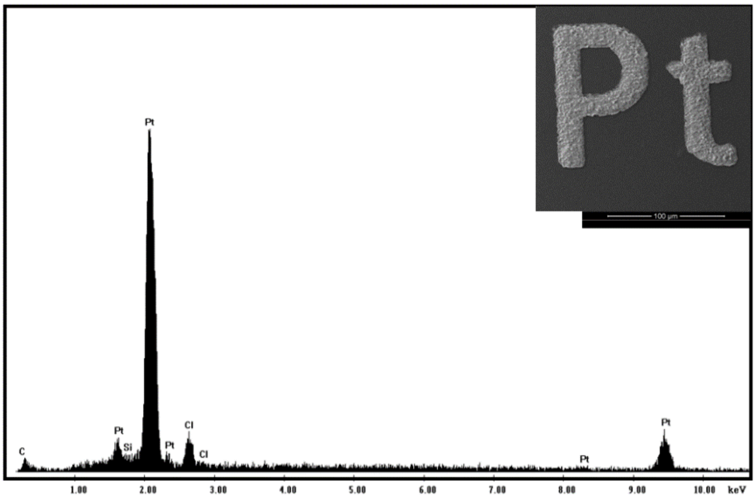

Figure S16. EDX of the deposited platinum structure

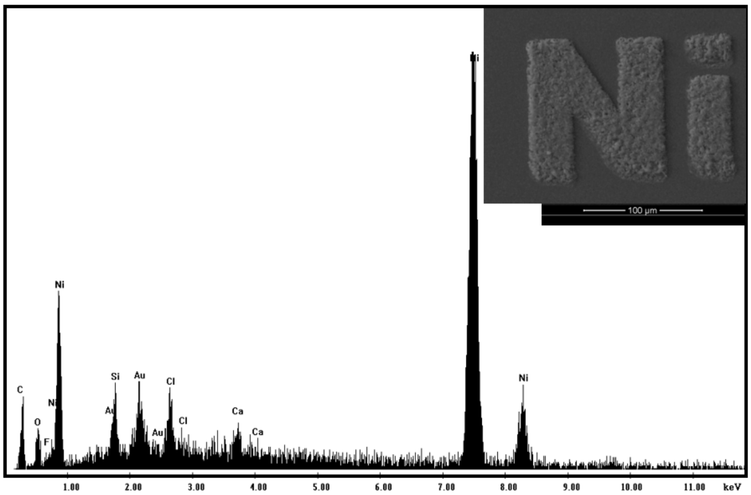

Figure S17. EDX of the deposited nickel structure

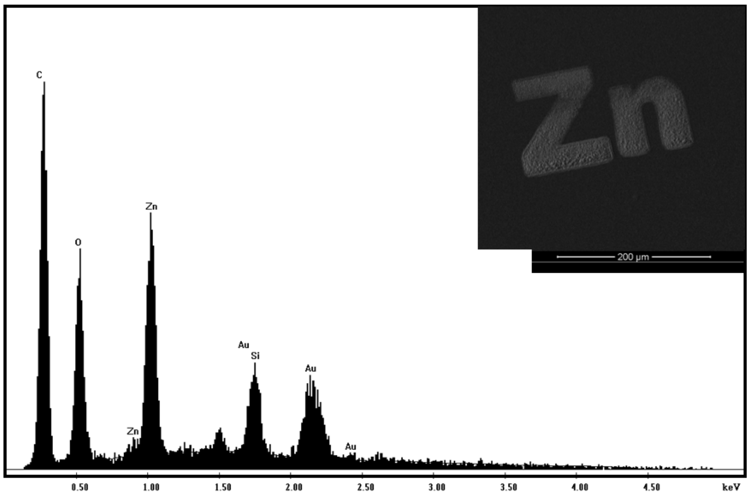

Figure S18. EDX of the deposited zinc structure.

(6) Surface quality of the deposited Fe line

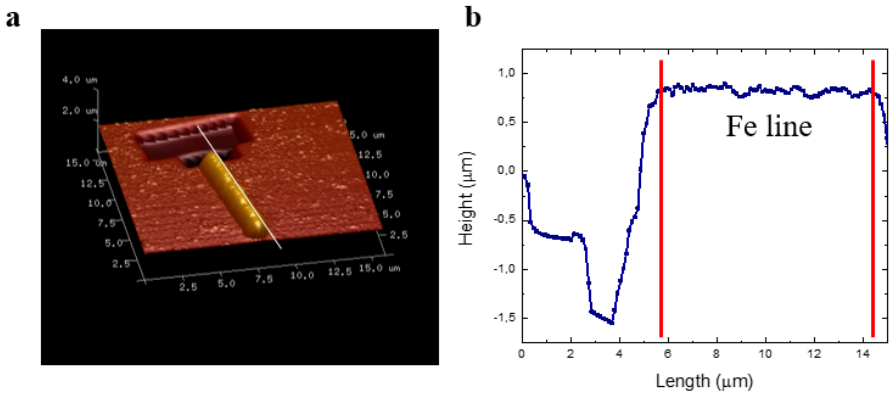

*Figure S19. **a**, Atomic force microscopy (AFM) image of the deposited Fe line. **b**, The line cut cross-section of the Fe line.*

The topography of the deposited Fe line (Fig. 3(a) in the main text), was characterized by atomic force microscopy (AFM) (Veeco Nanoscope IVa) as shown in Fig. S19(a). We further plot line cut cross-section of the Fe line shown in Fig. S19(b). We can deduce the root mean squared (RMS) of the surface roughness to be ~30 nm.

(7) Reduce the composition effect introduced by semiconductor nanoparticle

As shown in Section 3 in this chapter and the theoretical model in the next chapter, the deposited material is a composite of the material to be deposited and semiconductor particles. The performance of the composite material may be compromised by the introduction of the

semiconductor nanoparticles compared with the pure metal. To optimize the finished quality, we study the relationship between the concentration of carbon in deposition and the conductance.

Conductivity vs. Carbon Concentration

In the experiment to vary the final concentration of carbon in the deposition, we mix carbon ink (Ultra-black, Nano Kiwa-guro Ink) of various dilution between 200 and 6000 times with 30 mmol/L H_2PtCl_6 and ethanol by 1:1:2 volume ratio. For each solution, we deposited a set of 4 Pt squares with 60 μm×60 μm size for EDX to determine Pt to C ratio and 5 Pt lines with 24 μm×700 μm size for resistance and conductivity measurements. We used 57.6 mW laser power and 30 ms exposure time as exposure parameters for all the structures.

The average conductivity of Pt lines/Conductivity of bulk Pt is plotted against the Pt/C atomic percentage ratio in the deposition shown in Fig.S20. The Pt conductivity increases monotonically with respect to Pt/C atomic percentage. From the plot, it can be inferred that the concentration of the semiconductor carbon nanoparticle affects the conductivity of deposited material. When the carbon concentration is high, too many semiconductor nanoparticles are trapped in the composite and thus reduce the conductance. Among the solution receipts of various carbon in concentration, the best Pt/C ratio we can achieve is about 1.25. Using the structure in Figure S25 and the DLS measurement data in Figure S3, the 1.25 ratio indicates the building block structure of Pt deposition is a 60-140 nm diameter carbon nanoparticle covered by a Pt shell of about 15-35 nm thickness (assume the density of Pt is 21.45 g/cm^3 and C is 2.26 g/cm^3). The thickness is about 2-4 times of the penetration depth of 532 nm light into Pt. It fits the model we present in Section 4 (9), and in Figure S25. The maximum metal deposition around each semiconductor particle is limited by the light penetration ability. Therefore, there is an upper limit for the achievable Pt/C atomic percentage ratio under fixed exposure parameter.

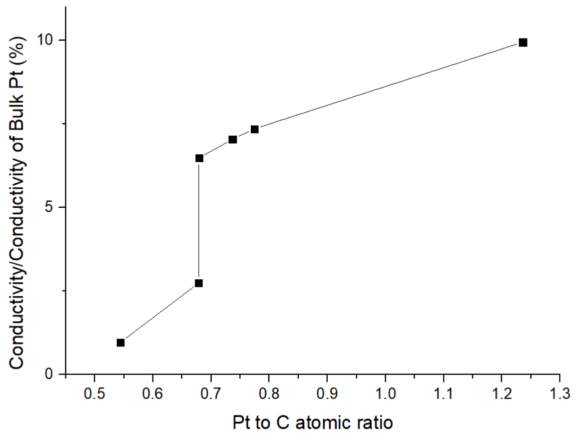

Figure S20. The conductivity of deposition depends monotonically on Pt/C ratio

Optimization of Iron Oxide insulator

Improved insulation quality

A similar protocol can be used for optimizing the iron oxide insulator. A 60 µm x 600 µm iron oxide composite line was deposited on a glass slide, using 50 mmol/L $FeCl_3$, 1000 times diluted carbon ink and ethanol with 1:1:2 volume ratio.

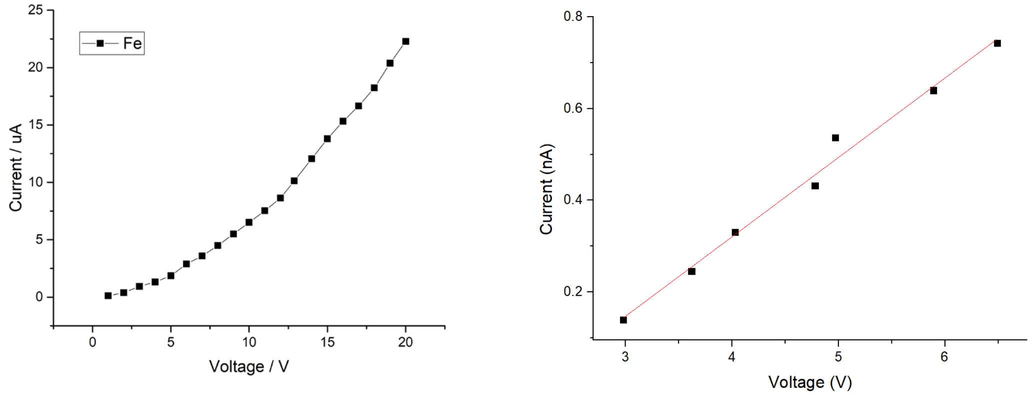

Figure s21. I-V curve of iron oxide. (Left) Use 100 times diluted carbon ink. (Right) Use 1000 times diluted carbon ink.

Figure S21 shows that using a more diluted carbon ink, the insulation quality can be significantly improved from MΩ region to GΩ region and the non-linear behaviour of the I-V curve is removed. A linear fit shows the resistance is about 5.7 GΩ. Using SEM and FIB, the length, width and height of iron oxide structure are measured to be 470.5 µm, 65.83 µm and 7.190 µm. The resistivity of our deposition is about $5.7\times 10^{5}\ \Omega \cdot cm$, which is on the same

order of magnitude compared with the micron thick iron oxide film synthesized by reactive magnetron sputtering [6].

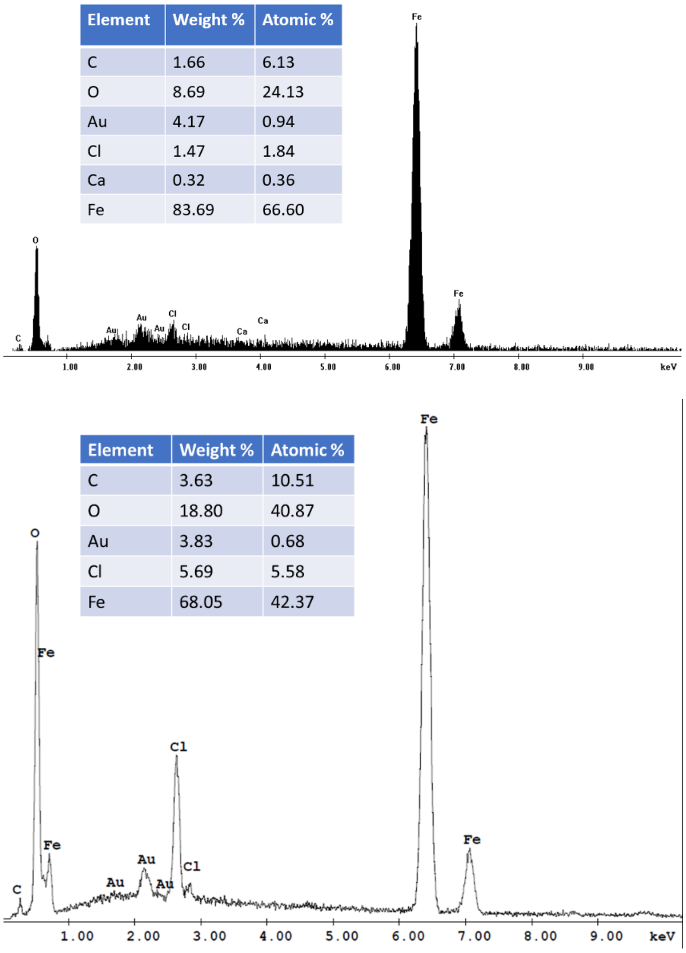

Element	Weight %	Atomic %
C	1.66	6.13
O	8.69	24.13
Au	4.17	0.94
Cl	1.47	1.84
Ca	0.32	0.36
Fe	83.69	66.60

Element	Weight %	Atomic %
C	3.63	10.51
O	18.80	40.87
Au	3.83	0.68
Cl	5.69	5.58
Fe	68.05	42.37

Figure s22. EDX of Iron oxide line made from (above) 100 times diluted carbon ink (below) 1000 times diluted carbon ink

In Fig.S22, the EDX shows that 10 times decrease of carbon ink concentration didn't change the carbon concentration in the iron oxide deposition significantly, but the Fe to O atomic percentage ratio is significantly improved from 2.74 to 1.04, indicating that much more portion of Fe is oxidized during deposition. Therefore, by optimizing the ink concentration, the deposition can be improved from the conductor (iron), insulator (iron oxide) and semiconductor (carbon nanoparticle) mixture towards pure insulator (iron oxide) structure with low carbon concentration.

Therefore, in summary for Pt and iron oxide deposition, both the insulation and conduction quality of deposition can be improved and controlled via optimizing the concentration of the carbon ink.

Improve the connection between each deposition pixel by sintering

Sintering

To further improve the performance of the deposited structures, the 1500 times diluted Japanese ink, 30mmol/L H_2PtCl_6 and ethanol were mixed by 1:1:2 volume ratio and 5 24 µm×700 µm lines were written on a glass slide. After deposition and cleaning with DI water, the Pt lines were sintered by the same laser with 30 ms exposure time and 60.4 mW laser power. Then EDX measured the Pt to C atomic percentage ratio of each line and length, width and thickness of each line were measured under SEM. The Pt to C atomic percentage ratio did not show any significant decrease compared to that of Pt lines before sintering, but the overall conductivity was improved from 10.70% to 17.39% of bulk Pt conductivity. The EDX results show that Cl disappeared after sintering.

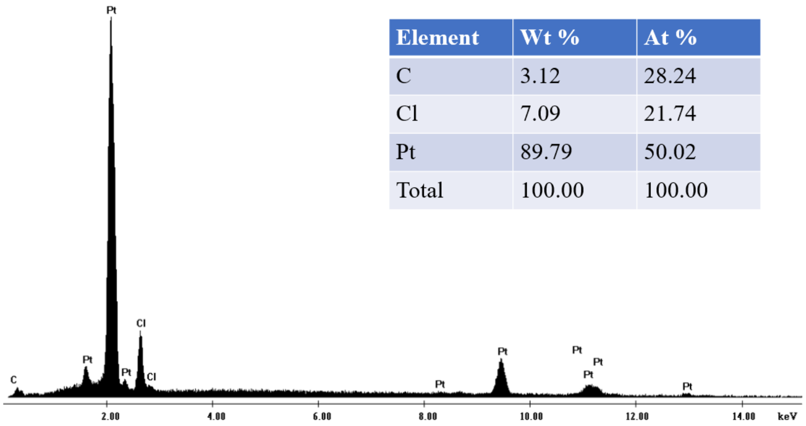

Element	Wt %	At %
C	3.12	28.24
Cl	7.09	21.74
Pt	89.79	50.02
Total	100.00	100.00

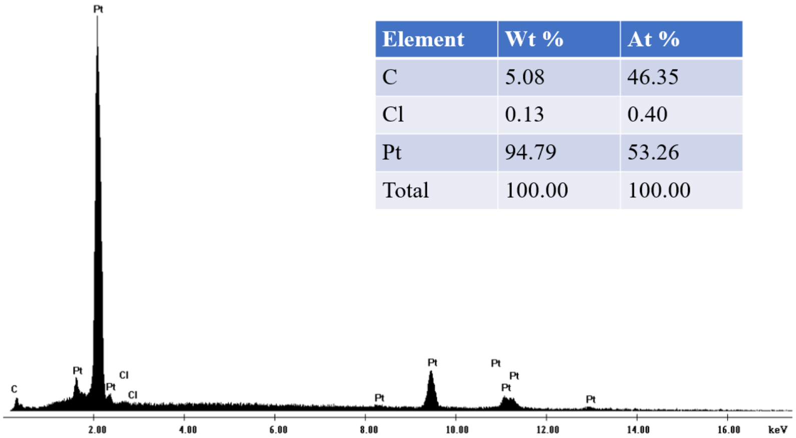

Element	Wt %	At %
C	5.08	46.35
Cl	0.13	0.40
Pt	94.79	53.26
Total	100.00	100.00

Figure S23. EDX of a Pt line without sintering (upper). There is a significant amount of Cl indicating the presence of incompletely reduced platinum chloride composite or chloroplatinic acid. EDX of a sintered Pt line (lower). A significant decrease in Cl concentration can be observed, while the Pt to C ratio remains almost unchanged.

The increase in conductivity might arise from improvement in the connection between adjacent Pt deposition pixel. The gap between deposition pixels may be filled by physically softened Pt due to heating by laser. It may also be filled by Pt particles chemically decomposed from unreduced H_2PtCl_6. It has been shown that when heated above 375 ℃, $H_2PtCl_6{\cdot}xH_2O$ gradually decomposes into Pt after several phases [7].

The presence of carbon after sintering shows there are carbon nanoparticles enclosed by Pt and isolated from air, so when the laser is focused upon deposition for sintering, carbon would not interact with oxygen but stay intact.

(8) Mechanical properties

Besides electrical properties, the mechanical properties are another important feature for deposited materials. One key issue is how elastic the material is. Without good bonding among components, the elasticity of the deposited material can be highly reduced and result in material failure during usage. The elasticity can be measured via the nanoindentation method [9].

Young’s/Elastic modulus measured by nanoindentation

A 175 µm X 175 µm Pt/C pad was deposited on a glass substrate. The nanoindentation was performed under Veeco diDimension icon AFM. The AFM probe is OMCL-AC240TS-R3 with 2 N/m spring constant from Olympus. The nanoindentation speed is 1 Hz with 2 µN indentation force. The deformation via applied indentation force was recorded during the indentation. A nanoindentation measurement was first performed on the glass substrate to calibrate the system.

The data are fitted using Hertz model as shown in equation (2),

$$P = \frac{2aEh}{1-\nu^2} \qquad (2)$$

where P is the loading force, a is the radius of flat-ended cylindrical punch (a=7nm), E is Young’s modulus, h is the indentation depth and ν is the Poisson’s ratio (Here we take Poisson’s ratio of graphite $\nu = 0.17$, because it turns out later in the measurement result that the carbon nanoparticle plays a critical role in mechanical property of the deposition). Between indentation force 0.29 µN and 1.29 µN, the indentation force and depth relation on the glass substrate is linear and the spring constant is fitted to be 2.00675 N/m. It is very close to the specified spring constant of the tip of 2 N/m. Therefore, it is reasonable to assume that the glass substrate is unindentable within this indentation force region. From the fitted spring constant, the deflection of the tip is subtracted from indentation measurement on Pt deposition within 0.29 µN to 1.29 µN indentation force region. A linear fit of the subtracted data gives Young’s modulus of deposition as ~1 GPa. It is the same order of magnitude of polyethylene (0.7 GPa), nitrocellulose (1.4 GPa), nylon (2.4 GPa), and graphite (5 GPa).

The deposited material contains graphite nanoparticles and Pt metals. The measured Young’s modulus is close to graphite, indicating this number is mainly determined by the graphite component as it is softer than the metals. On the other hand, at the same point of the

sample, multiple nanoindentations were performed to evaluate the reversibility of the deformation as plotted in the right graph of Fig. S24. Under 2 μN force (~13 GPa pressure) nanoindentation, the loading curves overlap with each other very well. The deposited structure was robust enough against such large indentation force, indicating that the deposited structures are mostly elastic.

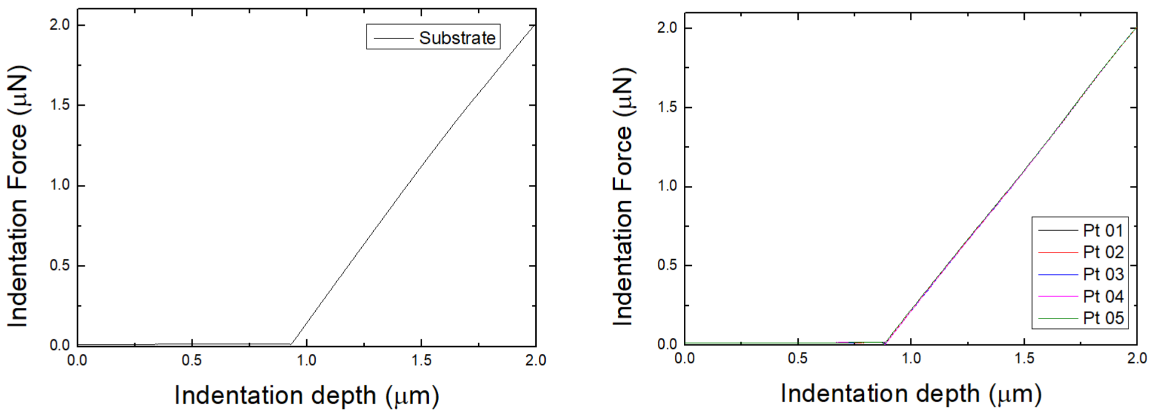

Figure S24. Nanoindentation measurement of Young's modulus. (Left) nanoindentation on a glass substrate for calibrating deflection sensitivity of cantilever. (Right) nanoindentation upon the same position on Pt pad was repeated 5 times and the overlapping of the 5 measurement shows the elasticity of deposition.

Flexibility

Inorganic structures usually are not flexible and may suffer from plastic deformation, especially for electrical gates made by noble metals. This is one of the difficulties of making flexible electronics with good conductivity. Here, we study the flexibility of this unique deposited composition.

Multiple indentations at the same position on a deposited Pt pad has shown its flexibility along with stress normal to the substrate surface. The overlapping feature among the 5 loading curves demonstrates the reversibility of deformation in the deposition. We also verify its flexibility along stress parallel to the substrate surface by bending our resistive flex sensor (a 50 μm × 300 μm Pt line on Kapton tape) to 1.12 mm radius of curvature and then return it to

flat state twice and check if it restores the initial resistance. The original resistance is 79.2 Ω and it becomes 78 Ω and 78.6 Ω after the first and second time bending respectively. It indicates that the deposition also demonstrates good flexibility along with horizontal stress.

Therefore, by combining nanoparticles with conductors, we realize a new material with the conductance comparable with noble metals while being flexible.

(9) Spatial resolution

There are three important parameters limiting the resolution of the LIMD. The size of the semiconductor particles, the penetration depth of the laser and optical diffraction limitation. Currently, the smallest carbon nanoparticles we found is from the Kiwa-guro Ink, ultra-black. The average size of the particle (D in Figure S25) is ~90 nm obtained by the dynamic light scattering measurements (see SI Section 3(1)). The penetration depth of laser (P in Figure S25) for the currently deposited materials is ranging from around 10nm (for conductor) to around 80 nm (for insulator). The optical diffraction limitation is roughly 200 nm-500 nm in our setup by using different objectives.

Currently, we can deposit 400-500 nm Pt nanoparticles. This is close to the theoretical limit.

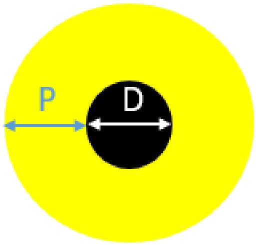

Figure S25 Scheme of nanoparticle deposited by the LIMD method. The centre black sphere is the carbon particle, with diameter D. The yellow shell is the Pt deposited on the surface of the carbon particle, with thickness of P.

Receipt for the solution (in volume ratio):

30 mmol/L H_2PtCl_6 : 1000 times diluted carbon ink : ethanol = 1 : 1 : 2

Exposure parameter:

Fixed: 532 nm laser, 1.3 NA and 100X magnification objective, oil, 2.68 mW

Result:

Using the lowest laser power possible for nucleation, multiple measurements are performed to demonstrate that for platinum using the current receipt, the achievable resolution is around 500 nm and this technique is capable of nanoscale lithography.

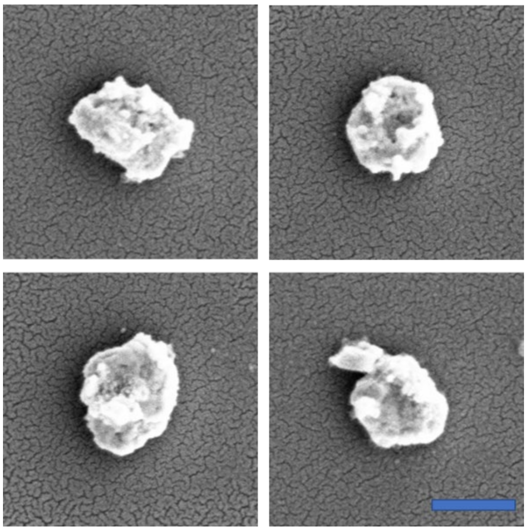

Figure S26. SEM images of smallest platinum dots obtainable so far with 2.68mw laser power. The blue scale bar is 500 nm.

Supplementary Note 5: Study of the mechanism of the LIMD method

(1) Model of deposition

The deposition is the combination of both optical trapping of the particles and the photo-induced chemical reduction. Nucleation and growth are involved in the deposition process.

In details:

1. Nucleation: The semiconductor particle moves towards the centre of the optical trap on the surface of the substrate. In the meantime, photo-induced reduction converts metal ions in the solution into the metal on the surface of the semiconductor particle. This reduction reaction also bonds the particle to the surface of the substrate.
2. Growth: More semiconductor particles move towards the optical trap. The reduction reaction bonds particles together with the metal layers.

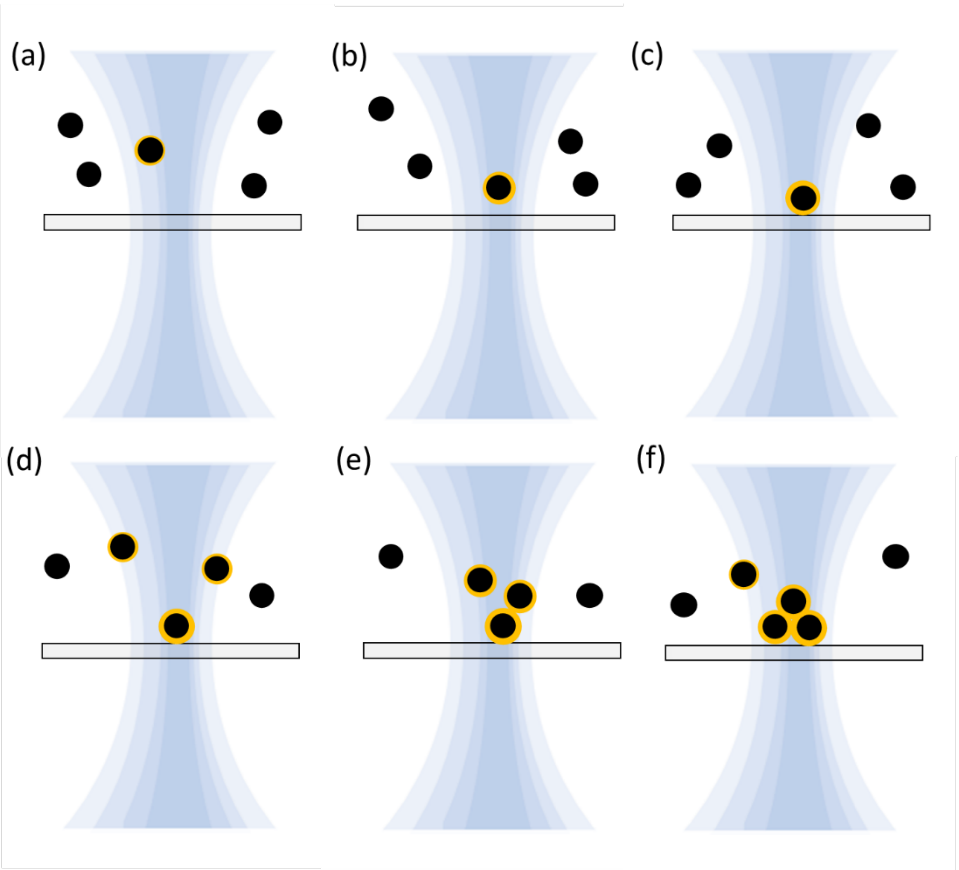

Figure S27. (a)-(c) Nucleation process. (d)-(f) Growth Process. (a) A carbon nanoparticle enters the optical trap and metallate is reduced to a thin layer of metal covering the carbon nanoparticle. (b) The carbon nanoparticle moves towards a position with higher laser intensity and since the focus is positioned on the substrate, the particle moves towards the substrate. (c)The metal layer grows thicker and the carbon nanoparticle falls onto the substrate and bonds to it. (d) More carbon nanoparticles enter the optical trap. (e) Carbon nanoparticles start to bond to each other via the metal layer covering them. (f) The deposited carbon nanoparticles occupy the part of the optical trap with highest laser intensity and it becomes less and less likely for other carbon nanoparticles coming later to be caught by the more and more distorted optical trap.

To verify this model, we perform several experiments.

Pure metallic solution and laser illumination would not induce deposition under the experimental conditions.

We verify this by the follow test:

Receipt for solution (in volume ratio):

(a) 30mmol/L H_2PtCl_6 : DI water = 1 : 1

(b) 1000 times diluted carbon ink : DI water = 1 : 1

(c) 1mg/L rGO : DI water = 1 : 1

(d) 30mmol/L H_2PtCl_6 : 1mg/L rGO = 1 : 1

(e) 30mmol/L H_2PtCl_6 : 1000 times diluted carbon ink = 1 : 1

The alphabet order corresponds to the solution used in Figure S28 from (a) to (e) accordingly.

Exposure parameter:

Fixed: 532nm laser, 57.1mW, 0.75NA and 20X magnification objective, air

Result:

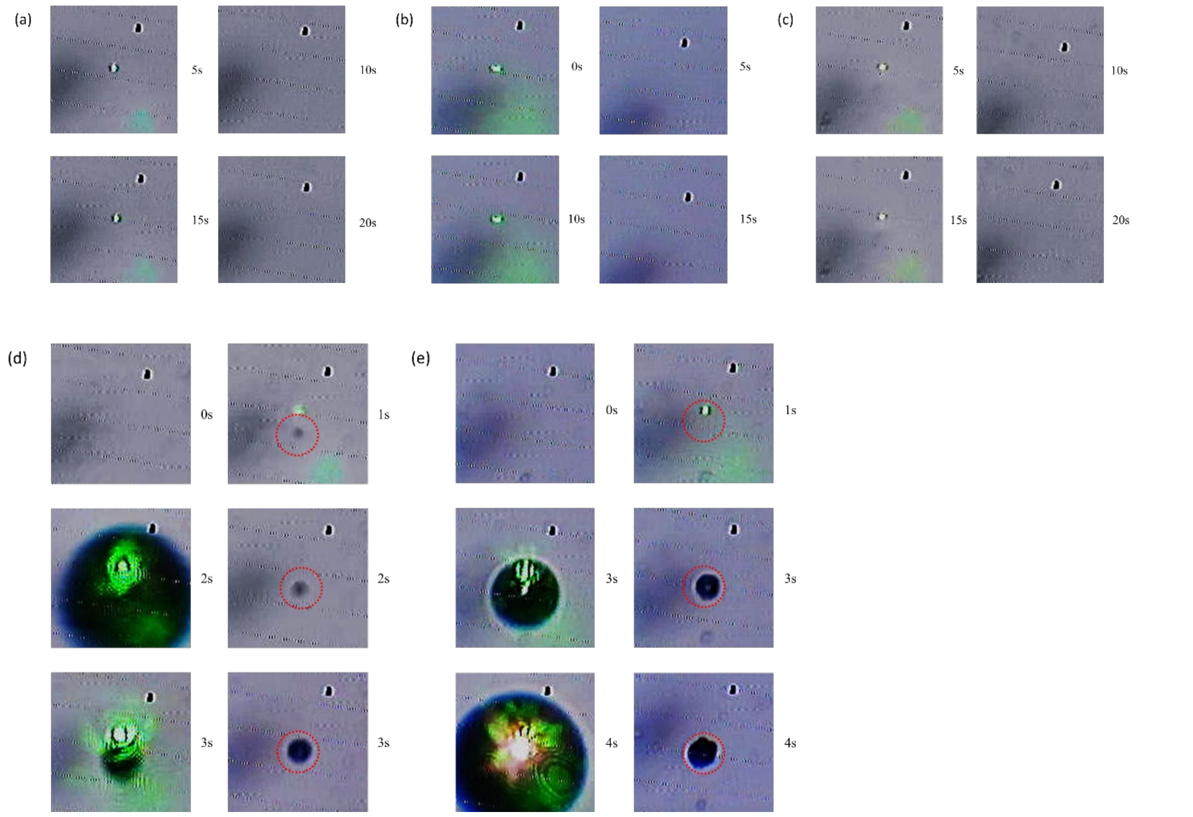

Figure S28. Screen shot of videos taken from transmission microscope CCD camera. The laser is constantly switched on/off to show deposition on the glass slide accumulates as the same position is continuously exposed by the focused laser beam. The red circle in (d) and (e) highlight the position where deposition happens under laser illumination.

The deposition process is monitored and recorded by a transmission microscope CCD camera (Figure S1) and some screen shots of videos are presented in Figure S28. When only metal solution or carbon nanoparticle solution (carbon ink or rGO) is used, a focused laser beam would not induce any deposition upon the substrate after long illumination period. When the two parts are mixed, the same focused laser beam would induce deposition at the focus

position almost right after switching the laser on. The transmittance images are recorded with the laser off. The black spots in Fig. 28 (d) and (e), indicates that materials were deposited (with the reducing of the transmission of the light) after the laser irradiation. As the laser beam is continuously applied to the same position, the area with deposition expands uniformly outward.

From this measurement, it is shown that the semiconductor particle is a key ingredient in this method. Laser power dependence can reveal more details of the mechanism. We separate the whole process into two parts, the nucleation and the growth.

(2) Wavelength dependence

Photo-induced reduction reaction requires the photon energy to be higher than the energy barrier. Thus, to verify this reaction is the mechanism, a laser wavelength dependence is needed. We tested material deposition under 3 light sources with different wavelength for this experiment.

Wavelength dependence of Au deposition with 532nm, 785nm, 840 nm laser.

Receipt for the solution (in volume ratio):

2.5mmol/L $HAuCl_4$: 1000 times diluted carbon ink : DI water = 1 : 1 : 2

Exposure parameter:

(a) 532nm laser, 4.48mW, 1.3NA 100X magnification objective, oil

(b) 785nm laser, 7.22mW, 1.3NA 100X magnification objective, oil

(b) 840nm laser, 10.0mW, 1.3NA 100X magnification objective, oil

Result:

Under the 532 nm wavelength laser irradiation, the deposition of Au/C material happens just 5 s after switching on the laser. On the other hand, similar experiments had been performed with 785 nm and 840 nm wavelength laser. After 2min illumination, no deposition can be observed on the CCD camera. The photon energy which is sufficient to reduced Au^{3+} to Au^0 is 1.69 eV. When the irradiation photon energy is lower than the threshold (1.69 eV), no deposition has been observed. Therefore, the deposition process is verified as a photo-induced reduction reaction.

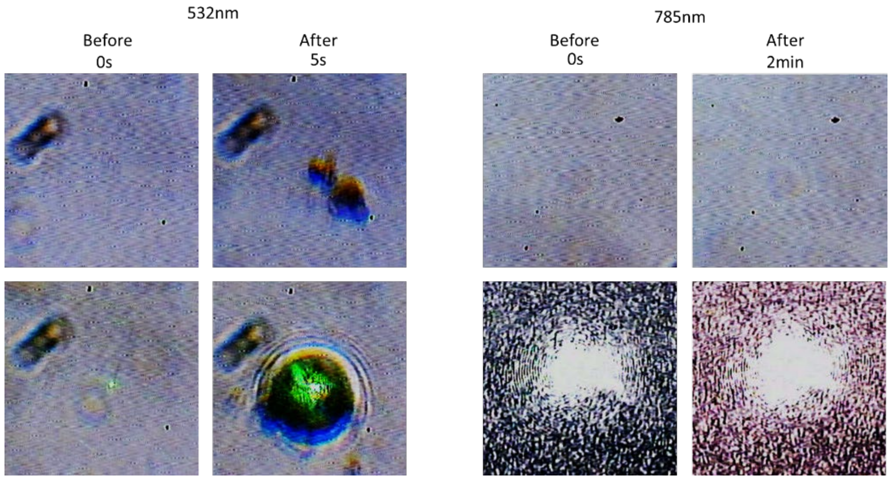

Figure S29. Screen shot of recordings by transmission microscope CCD camera. Each column of pictures consists of one with laser off and one with laser on at the same moment.

(3) Laser power and chemical concentration dependence

1. Nucleation

Nucleation Time of Gold

Receipt for the solution (in volume ratio):

2.5 mmol/L $HAuCl_4$: 1000 times diluted carbon ink : ethanol = 1 : 1 : 2

Exposure parameter:

Fixed: 532 nm laser, 1.3 NA and 100X magnification objective, oil

Variable: Laser power (measured in front of the objective)

Result:

The nucleation moment is judged based on whether Rayleigh scattering images showed up on the CCD camera. For high laser intensity irradiation, nucleation happens instantaneously after switching the laser on and nucleation time is hard to be determined by this method. For low laser intensity irradiation, the nucleation becomes slower, which is measurable. Under each laser power, nucleation time is measured 4 times.

In Figure S30, a reciprocal fit is performed using equation (3). T is the nucleation time, P is the laser power. N, α and T_0 are fitting parameters, where N is the total photon energy required for each nucleation, T_0 is the time measurement offset introduced by manually switching off the laser when nucleation happens, α is meant to check whether the relation is truly reciprocal.

$$T = \frac{N}{P^{\alpha}} + T_0 \qquad (1)$$

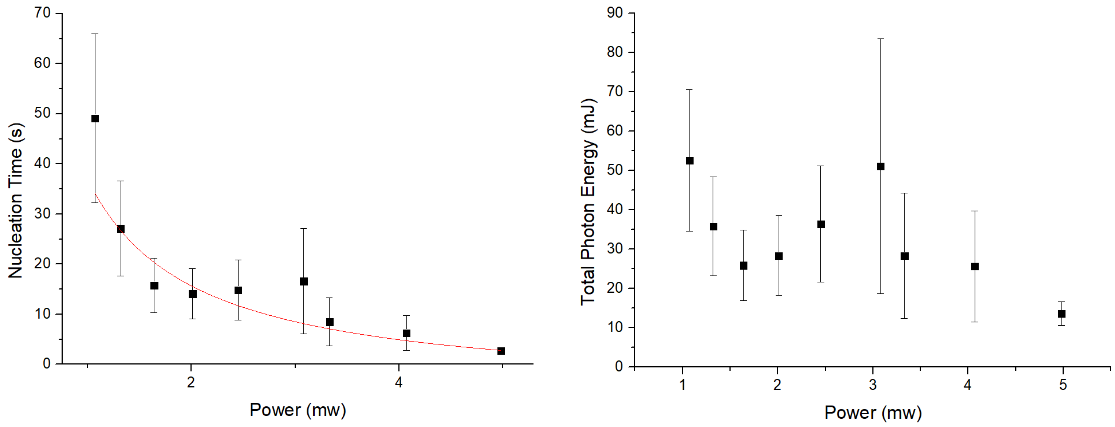

Figure S30. Au nucleation time vs power (left). Au total photon required for nucleation vs laser power (right).

The fitting result is that α is 0.99, very close to 1, which indicates that the total photon required for nucleation is a constant without clear power dependence, a good agreement with the photo-induced reduction reaction mechanism. This feature can also be verified by plotting the product of nucleation time and laser power vs laser power, the fluctuation shows no clear dependence of total photon required on laser power.

2. The growth after Nucleation

Laser power dependence of growth rate

Receipt for the solution (in volume ratio):

50 mmol/L $FeCl_3$: 1000 times diluted carbon ink : ethanol = 1 : 1 : 2

Exposure parameter:

Fixed: 532 nm laser, 1.3 NA and 100X magnification objective, oil

Variable: Laser power (measured in front of the objective), exposure time

Result:

Particles were deposited with different laser power and exposure time. SEM and FIB are used to measure the volume of each dot by assuming a sphere shape of deposition. Laser power for growth measurement is selected to obtain a good time control so that the size would not saturate immediately after nucleation and exposure time steps could be long enough. For each laser power and exposure time, the experiment is repeated 3 times.

Figure S31 clearly shows that the size of deposition increases with laser power and exposure time, while the growth rate per unit power does not depend on the power and decays as the deposition gradually grows outside the optical trapping range.

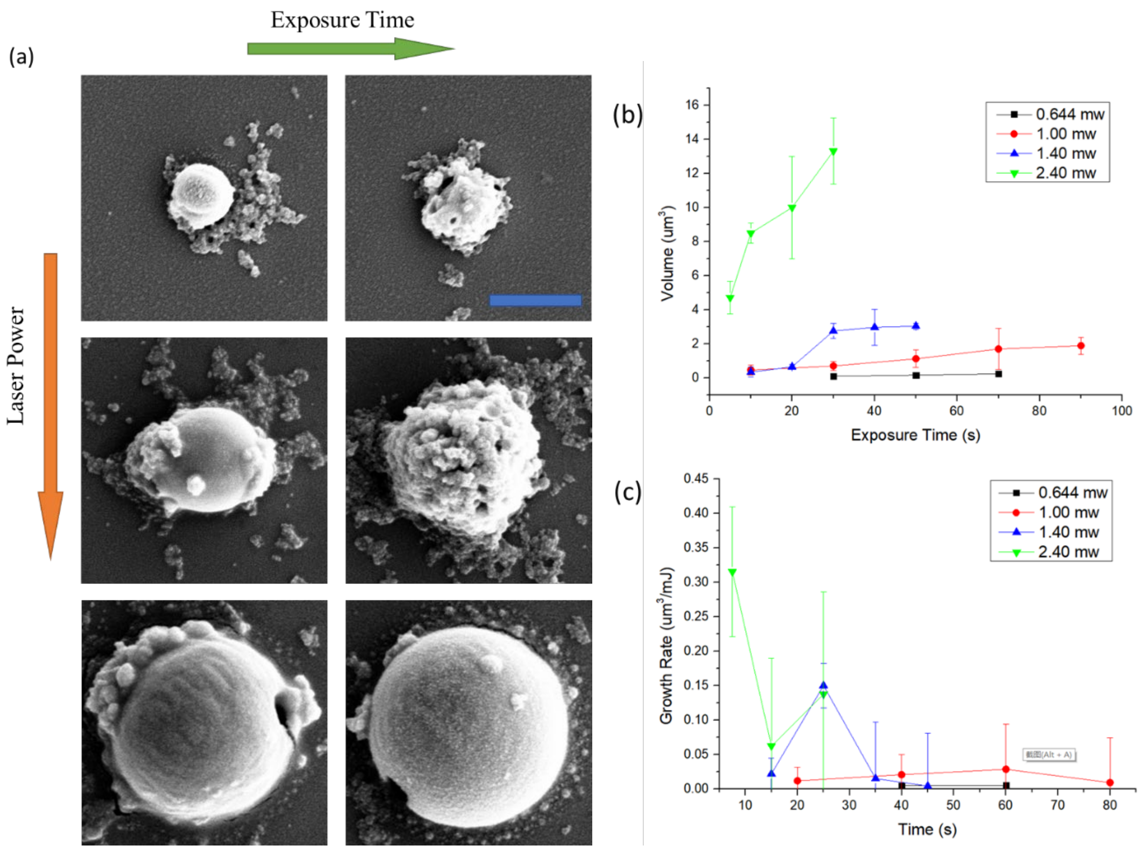

Figure S31. (a) SEM image of Fe deposition under different power and exposure time. The SEM image shows a half-sphere shape of deposition, so the volume of deposition can be approximated by just measuring the diameter to get the half-sphere volume. (b) Deposition volume vs Exposure time under different power. (c) Growth rate vs growth time under different power.

Carbon ink concentration and Salt concentration dependence of growth rate

Receipt for the solution (in volume ratio):

50 mmol/L $FeCl_3$: 1000 times diluted carbon ink : ethanol = 1 : 1 : 2

20 mmol/L $FeCl_3$: 1000 times diluted carbon ink : ethanol = 1 : 1 : 2

50 mmol/L $FeCl_3$: 200 times diluted carbon ink : ethanol = 1 : 1 : 2

Exposure parameter:

Fixed: 532 nm laser, 1.3 NA and 100X magnification objective, oil, 1.30 mw

Result:

The particles were deposited with different Fe^{3+} concentration and exposure time. SEM and FIB are used to measure the volume of each dot by assuming a sphere shape of deposition.

With reference to 50 mmol/L $FeCl_3$ 1000 times dilution ink receipt, an increase in carbon ink concentration would result in a higher growth rate and saturation size, while a change in salt concentration only affects the growth speed slightly without change to the saturation size.

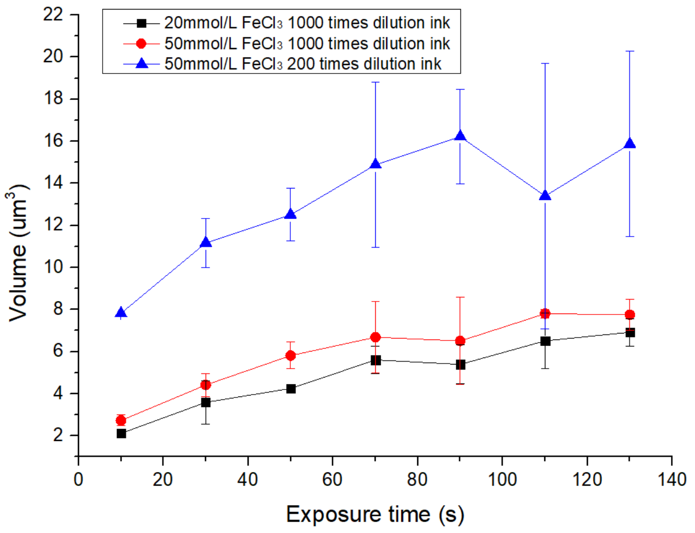

Figure S32. Growth dependence on ink concentration and salt concentration.

This measurement shows the optical trapping process plays a strong role in the mechanism. The "seeds", i.e., the semiconductor particles, limit the size of the structure.

As shown in Figure S32, the growth rate strongly depends on the ink concentration in the solution, and slightly depends on the metallate concentration. This is because the ink concentration is much less than the metallate. During the growth process, initially, when the size is still small, the speed of growth is nearly linear. In this stage, the centre of the optical trap is still occupied mainly by the liquid solution, not the deposited material, so the rate of ink particle going into the trap is a constant and the growth rate is linear. But when the size of the particle is bigger, it occupies a major part of the optical trap. Thus, the trap effectively becomes shallower. The higher the concentration of the ink particle in the solution, the higher the chance

that some ink particles can still fall into this trap as the total ink concentration is very low. Thus, the higher the ink concentration, the bigger the saturation particle size.

For comparison, the concentration of the metallate has nothing to do with the size of the saturated structure as it is limited purely by the trapping of the ink particle.

Furthermore, the optimized composition is also an important signature of the mechanism behind. Data in Note 4-7 show the optimized conductance occurred with atomic number ratio between Pt and C as nearly 1.2:1. This shows the role of the semiconductor particles as reaction centre and the metal as the glue.

(4) Analysis of the fine structures inside the deposition

If the growth rate is slow, the inner structure of the deposition is tightly packed, as shown in Figure S33.

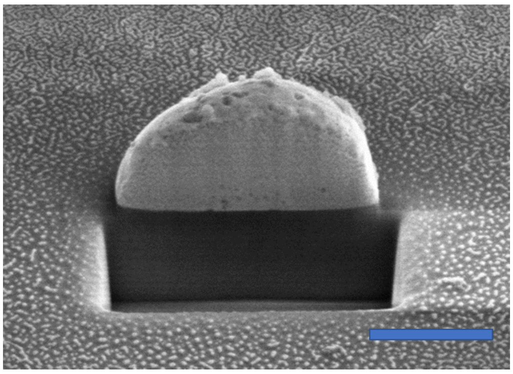

Figure S33. Cross-section of a Pt dot deposited under 4.05mw laser power using 1.3NA oil objective 15 s exposure time. The blue scale bar is 1 µm.

However, if the laser power is high and the growth rate is high, the inner structure of the deposition shows pillar like structure, as shown in Figure 3 c&d in the main text.

We notice that the width of these pillars does not differ much with different deposition materials, solution composition, the size of ink particles. As shown in Fig.S34, the width is around 170-190 nm for both Pt and iron oxides. This is because the width of the pillar is determined by the light penetration depth in the metal and the size of the semiconductor

particles. From the 532 nm light penetration depth listed in Table 2, the penetration depth is ranging from 10 nm - 80 nm. Together with 90 nm diameter carbon nanoparticles as growth centres, the total width should be around 130 - 250 nm, which agrees with our measurements in Figure S34.

Metal	Penetration Depth at 532nm
Pt	8.69nm
Au	12.3nm
Ag	12.0nm
Fe	7.49nm
Ni	8.61nm
Fe_2O_3	75.7nm

Table2. List of light penetration depth in various materials at 532nm (from https://refractiveindex.info/)

(a)

(b)

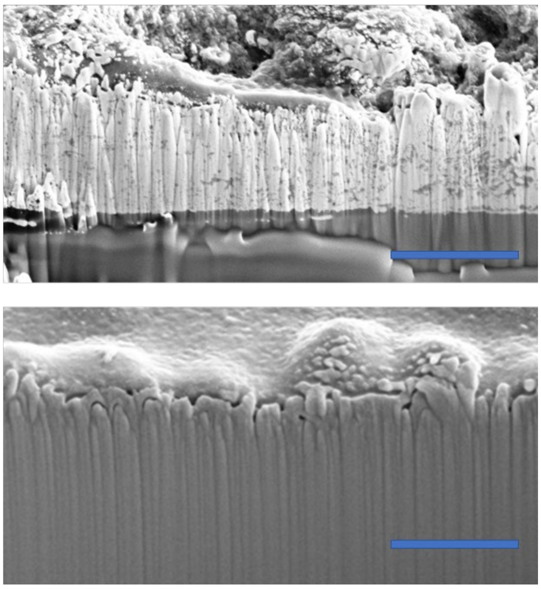

Figure S34. SEM image of the cross section of (a)Pt and (b) Iron-Iron oxide deposition. The width of several adjacent bars is first measured and divided by the total number of bars included in the measurement. The width of the bar in Pt deposition is 186 nm and in Iron-Iron oxide deposition is 169 nm.

Supplementary Note 6: Diamond quantum sensing

In this section, we use quantum sensing technique based on nitrogen vacancy (NV) centres in nanodiamond (ND) to study two distinct properties: 1. To study the potential heating effect

of the deposition. 2. To characterize the performance of deposited nickel and platinum microstructures[2-5].

The NV centre is a point defect in diamond crystal, which contains a substitution N atom directly connected to a vacancy with C_{3v} symmetry. The crystal axis between the nitrogen atom and vacancy defines the NV centre axis. There are 4 possible orientations in a single crystal diamond. Negatively charged NV^- centre is optically active, which shows the zero-phonon line at 637 nm with broad phonon sidebands. Both ground and excited states show a zero-field splitting which lifts off the degeneracy between m_s=0 and m_s=±1 spin states. The zero-field splitting of the ground spin state is 2.87 GHz. The Hamiltonian of NV^- can be described as:

$$H_{NV} = D\left(S_z^2 - \frac{S(S+1)}{3}\right) + \gamma \boldsymbol{B} \cdot \boldsymbol{S} + E(S_x^2 - S_y^2),$$

where D is the longitudinal component of the zero-field splitting, determined by both the temperature and the pressure. Under the same pressure, the change of D versus temperature follows dD/dT = -74.2(7) kHz/K, in the range from 0 ^{0}C to 100 ^{0}C. Thus, it can be used as a local temperature sensor.

E is the transverse component and works as an indicator of strain. The second term is the Zeeman term. In the reference frame of the NV centre, the longitudinal component along the NV centre axis $\gamma B_z S_z$ of the $\gamma \boldsymbol{B} \cdot \boldsymbol{S}$ term leads to the Zeeman splitting (here $B_z = B_{Long}$). The transverse component $\gamma(B_x S_x + B_y S_y)$ causes an overall frequency shift $\Delta\varepsilon \sim \frac{\gamma^2(B_x^2+B_y^2)}{D} = \frac{\gamma^2 B_{Trans}^2}{D}$.

The zero field splitting and Zeeman splitting term can be measured via optically detected magnetic resonance (ODMR) [reference 15-18 in the main text].

(1) The LIMD and Diamond quantum sensing correlated setup

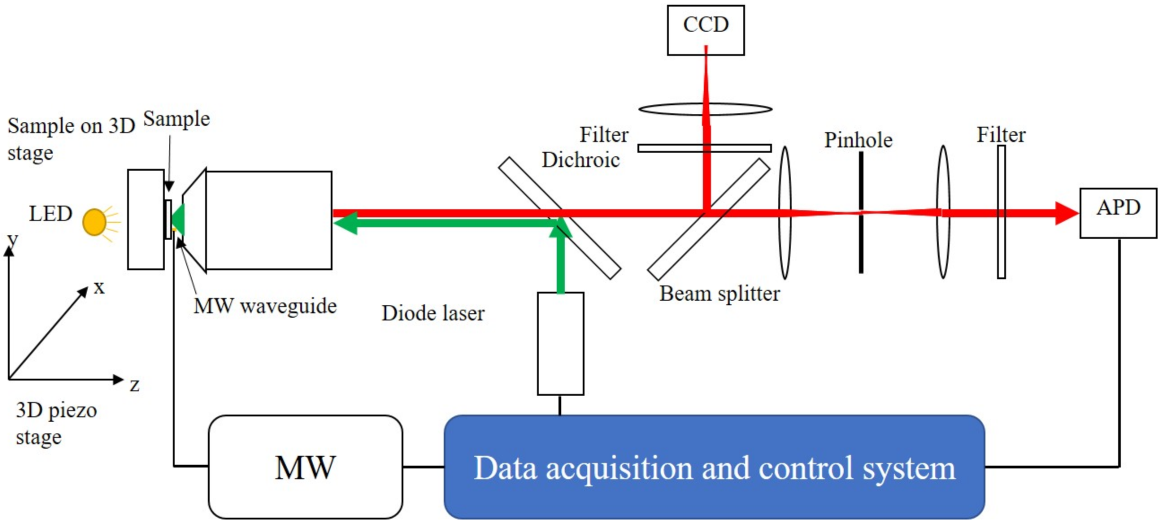

Figure S35. Experimental setup of the LIMD and the diamond quantum sensing dual-experiments. MW: Microwave generator. APD: avalanche photodiodes.

The experimental platform is an upgrade from the setup shown in Fig. S1. It is combined with a home-made optical confocal microscope (Fig. S35). Confocal microscopy is the basis of various optical measurements and platform for quantum information science based on solid state qubits. One of the advantages of the LIMD method is its compatibility of both setups. Thus, it can be used in-situ with optical measurements.

The experiments were performed under ambient conditions. The laser excitation was provided by a 520 nm laser diode. The phonon sideband emission was detected by Perkin-Elmer avalanche photodiodes (APDs). The microwave source was Rohde & Schwarz SMIQ signal generator with an amplifier (ZHL-16W-43-S+ from Mini-Circuit) to obtain the ESR spectra. In all the experiments, single crystalline NDs with an average size of 140 nm and a nitrogen concentration of 3 ppm from Ad´amas Nanotechnologies were used. On average each nanodiamond contains around 1200 NV centres.

(2) Sensing the potential heating during the deposition process

Photo-induced chemical reduction reaction may lead to the generation of heat due to non-radiative decay. Heating can be dangerous for the substrates. Thus, it is necessary to calibrate this heating effect during the deposition. Since the reaction happens in the tiny vicinity of

semiconductor nanoparticles, a local temperature sensor with high spatial resolution, close vicinity to the reaction centre and good temperature resolution is needed.

We spread nanodiamond particles containing NV centres on a glass substrate. We then use the LIMD method to deposit materials on nanodiamond surfaces and monitor the temperature change with NV centres *in-situ*. This is also a good demonstration that the LIMD method is compatible with optical measurements, thus, it can be used in parallel with them.

Methodology

The zero-field splitting D of NV^- centre responds linearly to temperature change from 0 ^{0}C to 100 ^{0}C by -74.2kHz/K. We measure the temperature shift of D by the offsets of the whole ODMR spectrum.

Since both laser excitation and microwave pulses can heat the system, both effects have to be calibrated in advance. To calibrate the diamond nanothermometer, the nanodiamond is immersed in DI water. By measuring the zero-field splitting D under 1.4 mW and 18.4 mW laser power, the heating effect brought by the increasing of the laser illumination power is obtained. It should be noted that despite the presence of heating effect brought by microwave power, it is actually decoupled from the measurement since only the D shift between two different laser power for the same nanodiamond is considered and both microwave source power and distance of nanodiamond from the waveguide is kept constant throughout the measurement.

After the calibration, to measure the temperature shifts in the LIMD process, the solution is replaced by the receipt for Fe deposition (50mmol/L $FeCl_3$: 1000 time diluted carbon ink : ethanol = 1 : 1 : 2). The same measurement of D shift under 1.4mw and 18.4mw laser power is performed. The resulting D shift is a combination of heat due to laser illumination and deposition process.

Results

When a nanodiamond is immersed in DI water, the heating due to laser power increase is just about 4.3 K. When a nanodiamond is immersed in $FeCl_3$ solution, the temperature increased from 1.4 mW to 18.4 mW laser power is about 15.1 K. It indicates that the deposition process would result in a local temperature increase on the order of 10 K as shown in Fig. S36.

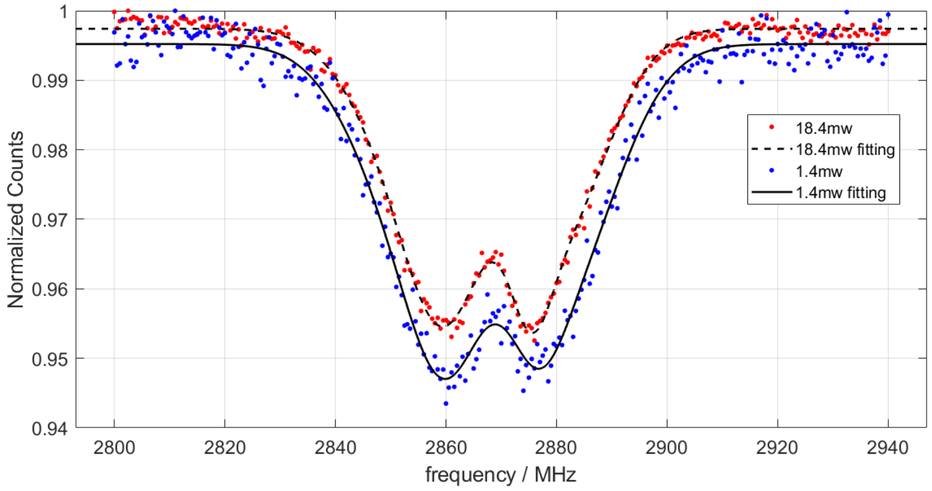

Figure S36. ODMR spectrum of 140nm nanodiamond immersed in FeCl3 solution under 1.4 mW and 18.4 mW 532 nm laser illumination. The D shift is approximately 1.12 MHz towards lower frequency, which corresponds to a local temperature increase of 15.1 K.

To be noted, this temperature change sensed by NV centres is a time-averaged effect. On the other hand, the laser excitation in this method is a CW laser. Thus, a big temperature jump is not likely to happen. It is still worth in the future to use other methods to study this temperature effect, for example, by measuring with transient absorption spectra with ultra-fast laser [8].

(3) Characterization of the magnetic profile of deposited Ni micro-magnets

After the deposition of Ni microstructures, the NDs were placed on the structure and their surroundings. 50 μm copper wire was placed next to the micro-structures to deliver MW. The ODMR measurement was applied to determine the magnetic field sensed by the NDs on top and aside from the nickel microstructures (see Fig. 3(h) (i) in the main text). The ODMR spectrum of the ND on the glass substrate (Fig. 3(i) in the main text) shows two resonance

frequencies, which is similar to the ODMR spectra of bare NDs without external magnetic field. The splitting between two resonances is due to the local strain E inside the particle. The ODMR spectrum of the ND on the nickel micro-structure (Fig. 3(h) in the main text) shows three pairs of resonance frequencies. From the outmost peaks, the ND sensed ~30 G magnetic field on the nickel micro-structure. This extra magnetic field is mainly contributed by the nickel micro-structure itself.

(4) Characterization of deposited Pt MW waveguides

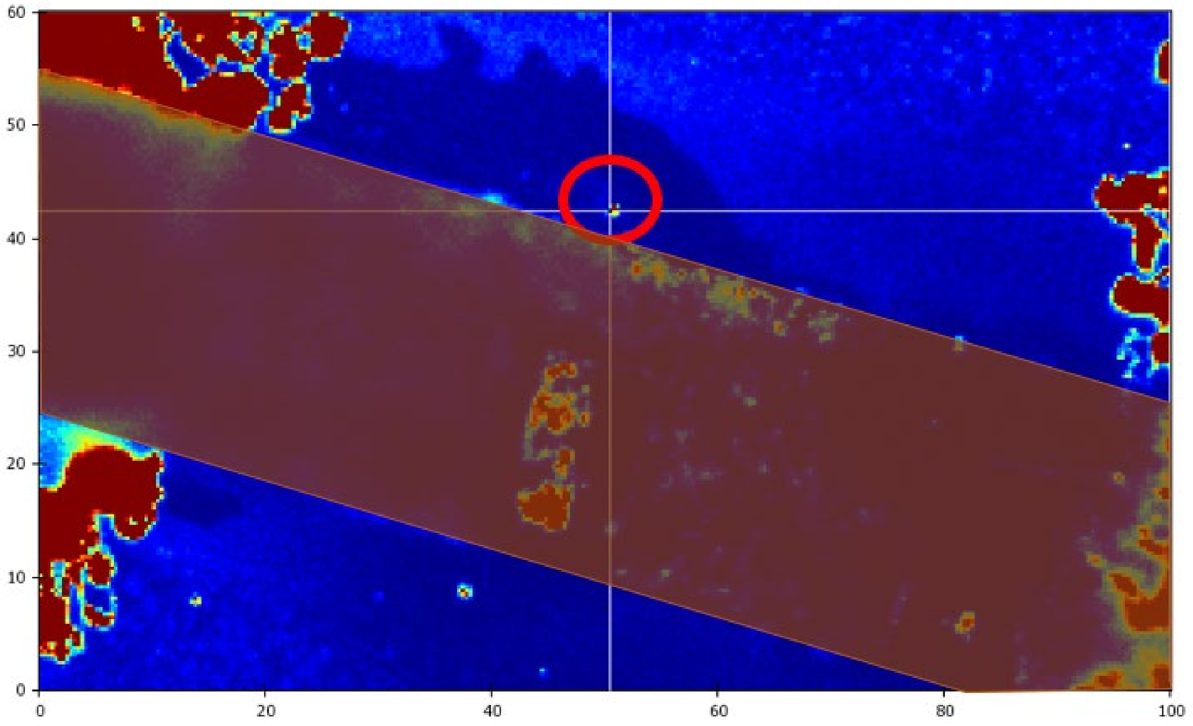

Figure S37. Fluorescence image of the deposited platinum MW waveguide. The measured nanodiamond next to the structure is marked with a circle.

In a two-level system, once an external electromagnetic field, which is on resonance with the two-level system, is introduced, this external field will induce oscillation of the population between the two-levels of the system. This is namely Rabi oscillation, indicating the ability to coherently control the two-level system. To explore NV centres in diamond as spin qubits, it is essential to coherently manipulate their electron spins.

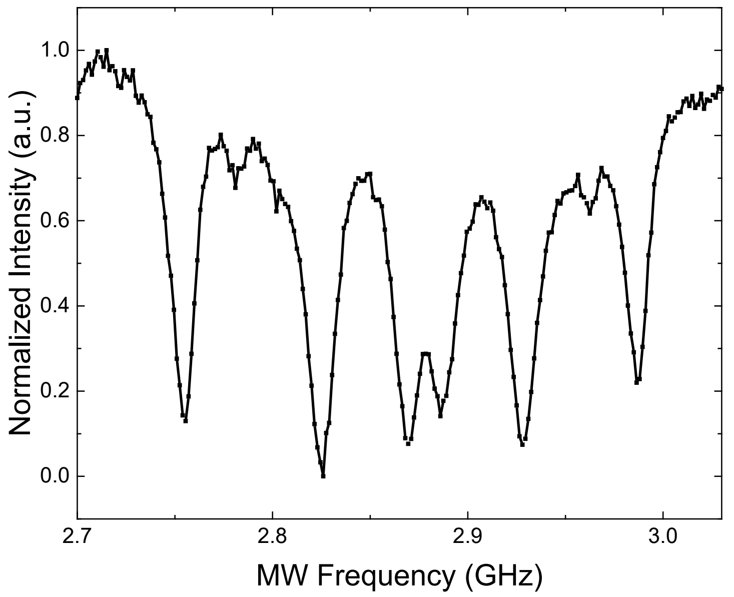

Figure S38. Optically detected magnetic resonance spectrum of the measured ND.

Figure S37 shows the fluorescence image of the ND next to the deposited Pt MW waveguide. An external magnetic field was applied to remove the degeneracy of the spin resonance frequencies. The ODMR spectrum of the measured ND is shown in Fig. S38.

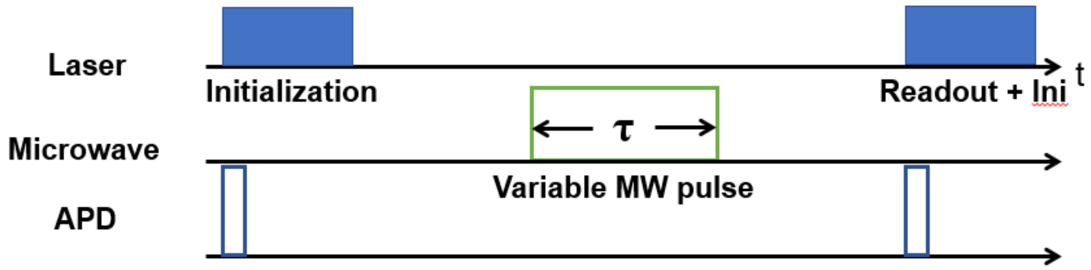

Figure S39. Experimental scheme and the pulse sequence of the Rabi oscillation measurements.

As soon as the Zeeman splitting of the ground spin states is determined by ODMR spectroscopy, a strong MW field can be applied on resonance with the spin transitions to drive the electron spin coherently. In the experiment, MW frequency was set on resonance with the left outmost peak (2.754558 GHz). It is shown in Fig. S39 that, a green laser pulse with 3000

ns pulse length was generated to initialize the NV spin state. After the optical pumping, the laser was turned off, and the MW source was switched on for a certain time to drive NV spins. When the coherent manipulation of electron spins was done, another green laser pulse with 3000 ns pulse length was generated to readout the population of the spin state, and initialize the NV electron spins, in preparation for the next measurement. The single-photon detector was gated to readout the NV fluorescence intensity during the first 200 ns of each laser pulse, to acquire good contrast.

Supplementary Note 7: Measurement on resistive flex sensor and touch sensor

(1) Resistive flex sensor

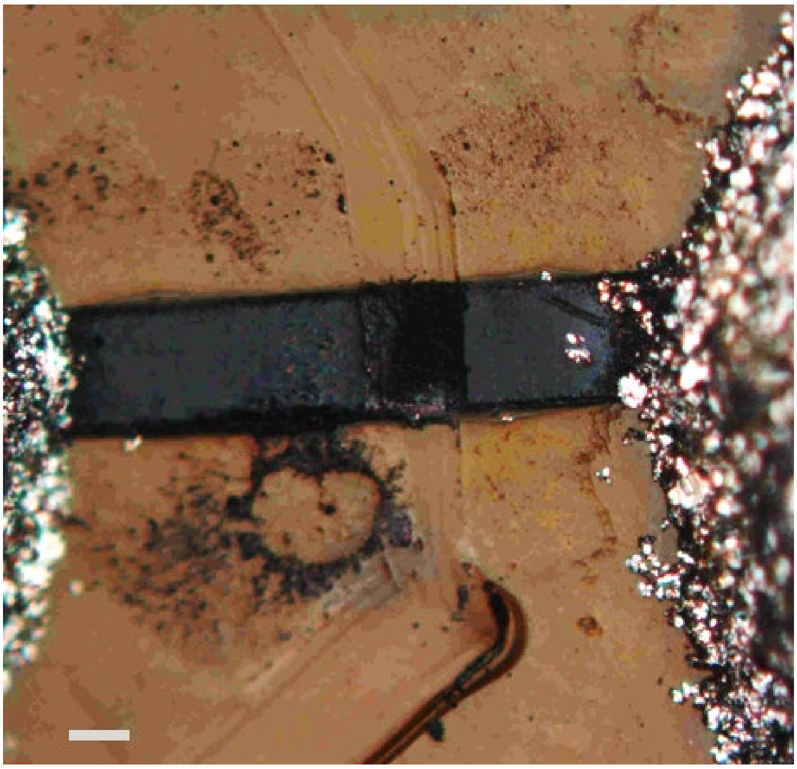

Figure S40. Optical image of the flex sensor. The middle part is the deposited platinum structure. It is contacted by silver glue on both sides. The substrate is Kapton tape. The scale bar is 25 μm.

The resistive flex sensor was created by printing a 300×50 μm platinum (Pt) line on the non-adhesive side of Kapton tape (see Fig. S40). The production of the sample followed the same

procedures mentioned previously. Copper wire was attached on the left and right end of Pt by silver glue (EPO-TEK H20E). A bendable plastic film together with a white paper was rolled to form cylinders with a various radius of curvature. The tape was then pasted on the plastic film. Copper wires were wrapped around probes connected to Keithley 2400 in constant current mode for resistance measurement and fixed by carbon tape (Adhesive Carbon Tape 8 mm × 20 m AGG3939) to ensure stable connection. The sample was pasted parallel and perpendicular to the curvature for resistance measurement along the Pt rectangle length and width respectively.

(2) Resistive touch sensor

The protocol is a 1D resistive touch sensor consisting of two 350 μm ×350 μm Pt squares 1.12 mm apart written on the non-adhesive side of the tape. The electrode for resistance measurement was made of silver glue (EPO-TEK H20E) and silver paste was used to connect copper wires and electrodes. Copper wires were wrapped around probes of Digital Multimeter (DMM FLUKE 79) and fixed by carbon tapes to ensure stable connection. The resistance of two Pt squares was monitored by two DMM simultaneously.

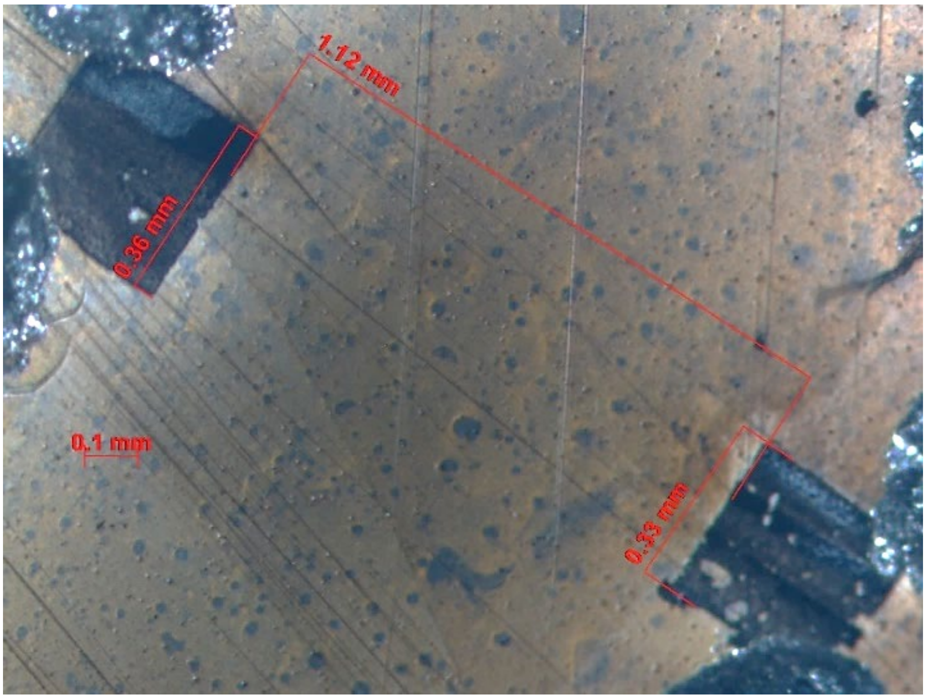

Figure S41 Optical image of the fabricated touch sensor. The substrate is Kapton tape. The scale bar is 100 µm.

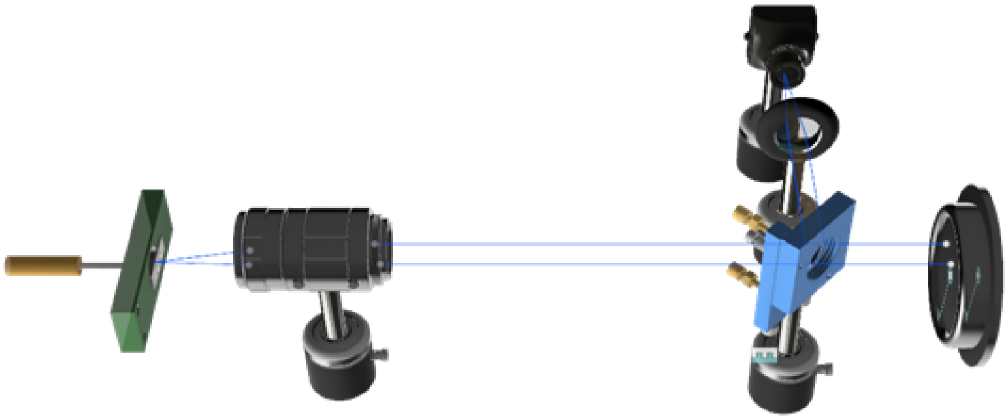

Figure S42 Schematic optical setup of the microscope

The optical setup was a microscope of 3.75X magnification. The PCB with the sample was mounted in front of the 10x objective (Olympus RMS10X). The image was focused with an f=7.5 cm plano-convex lens onto an IDS industry CCD camera. The tape was attached to a Printed Circuit Board (PCB) with an aperture at the centre where touch was introduced. To ensure a proper and reproducible touch, a needle tip was mounted on a 3D stage and a ball of solder was attached to the tip. By adjusting the 3D stage, the position and depth of touch could be well controlled. When the solder ball did not touch the tape, a clear picture of the Pt squares

and silver glue electrodes was observed using the industry CCD camera. When the solder ball just touched the tape, it would lead to a defocused image of the sample and a slight increase in the resistance reading of the DMM. From that position, the solder ball was moved forward by 200 μm so that at each position the degree of bending was about the same.

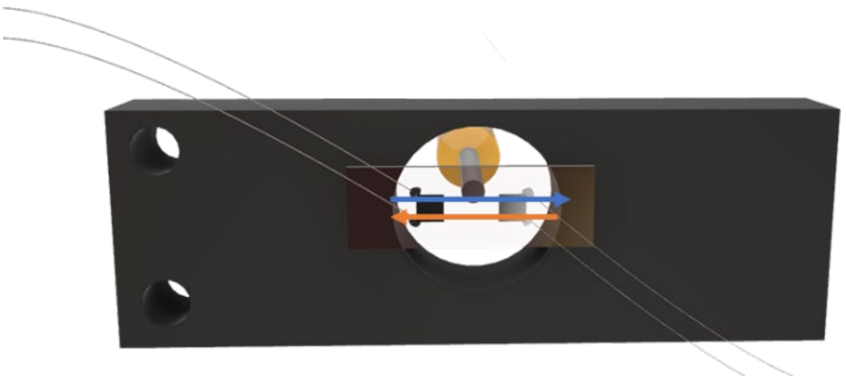

Figure S43. Schematic drawing of resistance measurement. Each Pt square had two silver glue electrodes with copper wires connected to a DMM and the tip behind was moved along the Pt squares to simulate touching different positions.

The data recording process was as the following: 1). The tip was first moved far away from the tape and resistance for each Pt square was recorded as R_0. 2)Then the tip was moved forward to the desired position and resistance was recorded as R_1. 3)The tip was moved away from the tape until a clear image of the sample recovered in the CCD camera and moved horizontally to the next desired position. By repeating steps 1) to 3), a set of R_0 and R_1 data pairs were obtained for each Pt square at each touchpoint ranging from the left side of the left square to the right side of the right square in both directions (blue and orange arrows in Fig. S43). By checking R_0, the robustness of Pt squares after each touch at different positions could be studied. In the plot of normalized R_1/R_0 against touch position, the different responses of two Pt squares with respect to touch positions showed this laser writing technique could in principle be applied to produce a touch sensor. Comparing the plot of two different tip motion directions (blue and orange arrow), the performance of the touch sensor was demonstrated to be reproducible.

Supplementary Note 8: Circuit board repair

Receipt for the solution (volumetric ratio):

30mmol/L H_2PtCl_6 : 1500 times diluted carbon ink: ethanol = 1 : 1 : 2

Exposure parameter:

532nm laser, 57.6mw, 0.75NA and 20× magnification objective, air

Experiment and Result:

The original ITO structure is a 14.9 mm long, 2 mm wide, 135 nm thick ITO thin film on a glass substrate, which initially has a resistance of 40.0 Ohm. Then using a diamond, a scratch is produced across the line structure to make it completely open circuit.

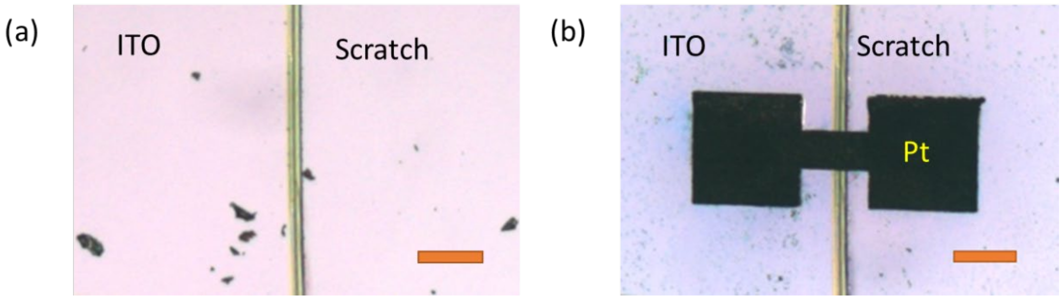

Figure S44. Bright field image of damaged ITO structure (a)before fix and (b)after the fix. The width of the scratch is around 20um. The orange scale bar is 100um.

The damaged ITO structure is sonicated successively in acetone, IPA and DI water for 15 minutes and plasma cleaned for 5 minutes to reduce the contact resistance between ITO thin film and Pt pad to be deposited. Then the damaged structure is mounted onto the xyz stage in Figure S1. The damaged region is located by manually moving it into the field of view via an xyz stage. And a bridge structure image is loaded for deposition (it consists of two 175 µm x 175 µm pads connected by a 60 µm x 120 µm line). The pads are meant to increase contact area and reduce contact resistance and the line is for connecting the two separated ITO regions.

The damaged structure turns from non-conductive to 53.9 Ohm, despite the uneven nature of the scratch. The whole scratch is about 2 mm long and the fixture covers only 60 μm length, if the entire scratch is filled by Pt, the ITO structure should return to its original performance.

We demonstrate that this laser writing technique can be applied for fixing micro malfunction in circuits. It should be possible to locate and fix circuit defects of size down to 5 μm, but since we did not have a good circuit sample or a method to deliberately introduce such a defect, the demonstration is about filling a 20 μm wide uneven gap between two conductive structures. The LIMD offers a powerful tool to repair microelectronics under complex environments, especially where other methods might not be suitable.

Supplementary Note 9: Reflow soldering

A 350 μm×350 μm Pt pad was written on a glass slide (see Fig. S45). The Pt pad was laser sintered after cleaning, to remove any carbon residue, to ensure the contact interface was Pt-solder paste-copper wire.

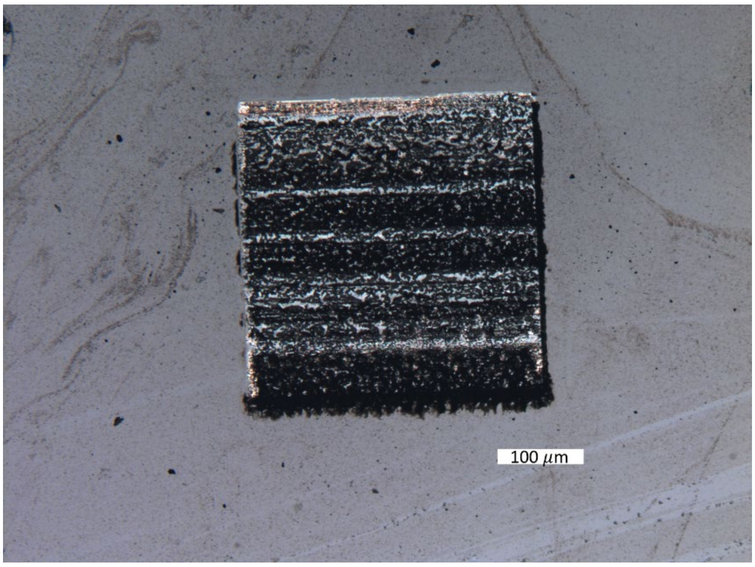

Figure S45. Optical image of the Pt pad before reflow soldering.

The solder paste (Chip Quik SMD291AX10) was dipped onto the Pt pad by a sharp needle. A bare copper wire of 50 μm diameters was laid straight across the Pt pad. The glass slide holding the Pt pad was put into an oven (Thermolyne Type 10600) following the recommended temperature profile.

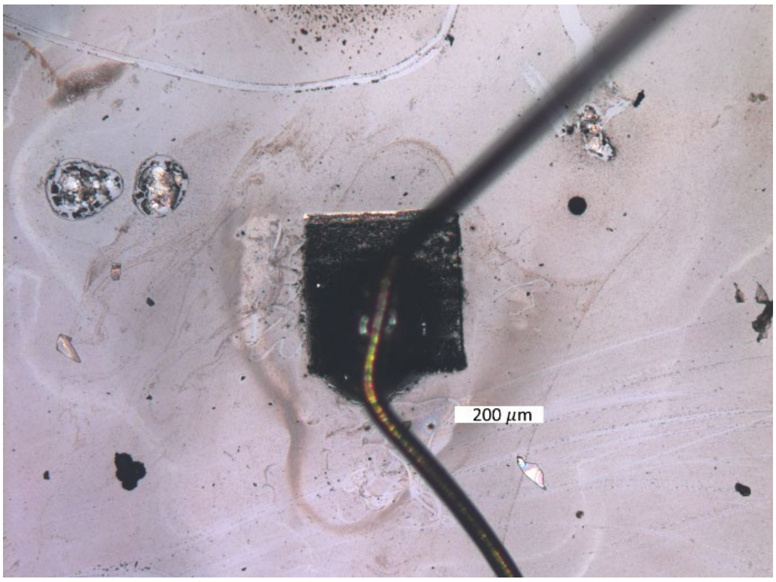

Figure S46. Pt pad after reflow soldering

After cleaning any flux residues, the copper wire is bent and lifted. The Pt pad remained well attached to the substrate, which indicates that this laser writing technique has the ability to produce structures, that can be utilized as landing pads for surface-mounted electronic components.

Supplementary References

1. Keithley, Tektronix, Low Level Measurements Handbook - 7th Edition

2. Jelezko, F. & Wrachtrup, J. Single defect centres in diamond: A review. *Physica status solidi (a)* 203, 3207(2006).

3. Balasubramanian, G. et al. Nanoscale imaging magnetometry with diamond spins under ambient conditions. *Nature* 455, 648 (2008).

4. Maze, J. R. et al. Nanoscale magnetic sensing with an individual electronic spin in diamond. *Nature* 455, 644 (2008).

5. Yip, K. Y. et al. Measuring magnetic field texture in correlated electron systems under extreme conditions. *Science* 366, 1355 (2019).

6. E. Aubry. et al. Synthesis of iron oxide films by reactive magnetron sputtering assisted by plasma emission monitoring. *Materials Chemistry and Physics* 233 (2019) 360-365.

7. A. E. Schweizer & G. T. Kerr. Thermal Decomposition of Hexachloroplatinic Acid. *Inorganic Chemistry*, Vol 17, No.8 (1978).

8. Tetsuro Katayama, Kenji Setoura, Daniel Werner, Hiroshi Miyasaka, and Shuichi Hashimoto, Picosecond-to-Nanosecond Dynamics of Plasmonic Nanobubbles from Pump−Probe Spectral Measurements of Aqueous Colloidal Gold Nanoparticles， Langmuir 2014, 30, 9504−951.

9. Bharat Bhushan. Depth-sensing nanoindentation measurement techniques and applications. *Microsyst Technol* (2017) 23:1595–1649.
